# Construction and Clinical Translation of Causal Pan-Cancer Gene Score Across Cancer Types

**DOI:** 10.3389/fgene.2021.784775

**Published:** 2021-12-23

**Authors:** Shiyue Tao, Xiangyu Ye, Lulu Pan, Minghan Fu, Peng Huang, Zhihang Peng, Sheng Yang

**Affiliations:** ^1^ Department of Biostatistics, Center for Global Health, School of Public Health, Nanjing Medical University, Nanjing, China; ^2^ Department of Epidemiology, Center for Global Health, School of Public Health, Nanjing Medical University, Nanjing, China

**Keywords:** pan-cancer, risk estimation, genome-wide association study, summary statistics, fine-mapping

## Abstract

Pan-cancer strategy, an integrative analysis of different cancer types, can be used to explain oncogenesis and identify biomarkers using a larger statistical power and robustness. Fine-mapping defines the casual loci, whereas genome-wide association studies (GWASs) typically identify thousands of cancer-related loci and not necessarily have a fine-mapping component. In this study, we develop a novel strategy to identify the causal loci using a pan-cancer and fine-mapping assumption, constructing the CAusal Pan-cancER gene (CAPER) score and validating its performance using internal and external validation on 1,287 individuals and 985 cell lines. Summary statistics of 15 cancer types were used to define 54 causal loci in 15 potential genes. Using the Cancer Genome Atlas (TCGA) training set, we constructed the CAPER score and divided cancer patients into two groups. Using the three validation sets, we found that 19 cancer-related variables were statistically significant between the two CAPER score groups and that 81 drugs had significantly different drug sensitivity between the two CAPER score groups. We hope that our strategies for selecting causal genes and for constructing CAPER score would provide valuable clues for guiding the management of different types of cancers.

## Introduction

Cancer is a major cause of mortality in both developed and developing countries, resulting in more than 8 million deaths each year worldwide (Tarver, 2012; Bray et al., 2013; Rodriguez-Martin et al., 2020). Since the causal factors and regulatory mechanisms are complex and remain largely unknown, there could be an increasing trend of morbidity and mortality attributed to cancer in the future (Nakagawa and Fujita, 2018). Based on multi-omics technologies, it has been demonstrated that cancers in different tissues and organs may share common features, whereas those in the same or similar organ may have distinct characteristics (Ciriello et al., 2013; Kandoth et al., 2013; Peng et al., 2021). These findings indicate that a pan-cancer strategy, which takes into account commonalities across cancer types, can be used to identify molecular abnormalities that transcend particular lineages, may explain oncogenesis, and make a large contribution towards the personal management of cancer (Vargas and Harris, 2016). In addition, pan-cancer analysis improves the statistical power used to identify cancer-related molecular dysregulation and avoids poor reproducibility in the characterization of rare subtypes (Priestley et al., 2019). Programs, such as the Cancer Genome Atlas (TCGA), which coordinate multi-omics sequencing and the clinical annotation of approximately 10,000 samples across over 30 cancer types, provide a great opportunity to identify pan-cancer biomarkers (Chang et al., 2013; Nawy, 2018).

Although genome-wide association studies (GWASs) have identified thousands of cancer-related loci (De Los Campos et al., 2018), there are still some unsolved issues. First, the majority of GWAS have identified variants located in non-coding regions and with small effect sizes, making it difficult to interpret functional and biological mechanisms that underlie the associations (Maurano et al., 2012; Visscher et al., 2017; Zeng et al., 2021). Second, complex linkage disequilibrium (LD) may obscure causal variants that drive the associations. Therefore, significant associations identified by GWASs are more about disease-related genomic regions than individual variants (Gallagher and Chen-Plotkin, 2018; Tam et al., 2019). Third, the most statistically significant variants may not be causal.

In general, genetic variants cause complex diseases by regulating gene expression, the abundance of one or multiple downstream proteins (Lappalainen et al., 2013; Westra et al., 2013; Albert and Kruglyak, 2015; Gusev et al., 2016). Gene pathway analysis and enrichment analysis have been widely applied to explore potential cancer-related mechanisms and have supplied plenty of valuable clues for the development of intervention targets (Shukla et al., 2016; Bao et al., 2019; Demircioğlu et al., 2019; Peng et al., 2019; Kim et al., 2020). Although previous studies have also leveraged data from TCGA to identify specific genes and signaling pathways involved in oncogenesis and development from a pan-cancer perspective (Ballot et al., 2020; Frost et al., 2020; Liu et al., 2020), these candidate genes or pathway-specific strategies are all based on prior knowledge, resulting in the loss of potential causal associations (Deng et al., 2014; Liu et al., 2014; Zhang et al., 2018). In addition, many of the associated genes identified may be the outcome rather than the cause of the disease (Gusev et al., 2016). Consequently, transcriptome-wide association studies (TWASs) were proposed to integrate GWASs with expression quantitative trait locus (eQTL) reference panels constructed from external genome-wide gene expression and genotype data to identify predicted gene–trait associations (Xu et al., 2017; Barbeira et al., 2018). Since a large sample size is used, the performance and statistical power of a TWAS is superior to that of traditional transcriptome analysis (Gusev et al., 2016; Xu et al., 2017; Barbeira et al., 2018). However, TWASs tend to identify multiple significant genes per region but fail to define the causal gene due to LD confounding (Mancuso et al., 2019; Wainberg et al., 2019; Wu and Pan, 2020). To overcome this drawback, fine-mapping methods were used to identify causal variants responsible for complex traits by accounting for the patterns of LD among the SNPs within a region associated with the target disease and assuming that at least one causal variant exists (Schaid et al., 2018).

**FIGURE 1:**
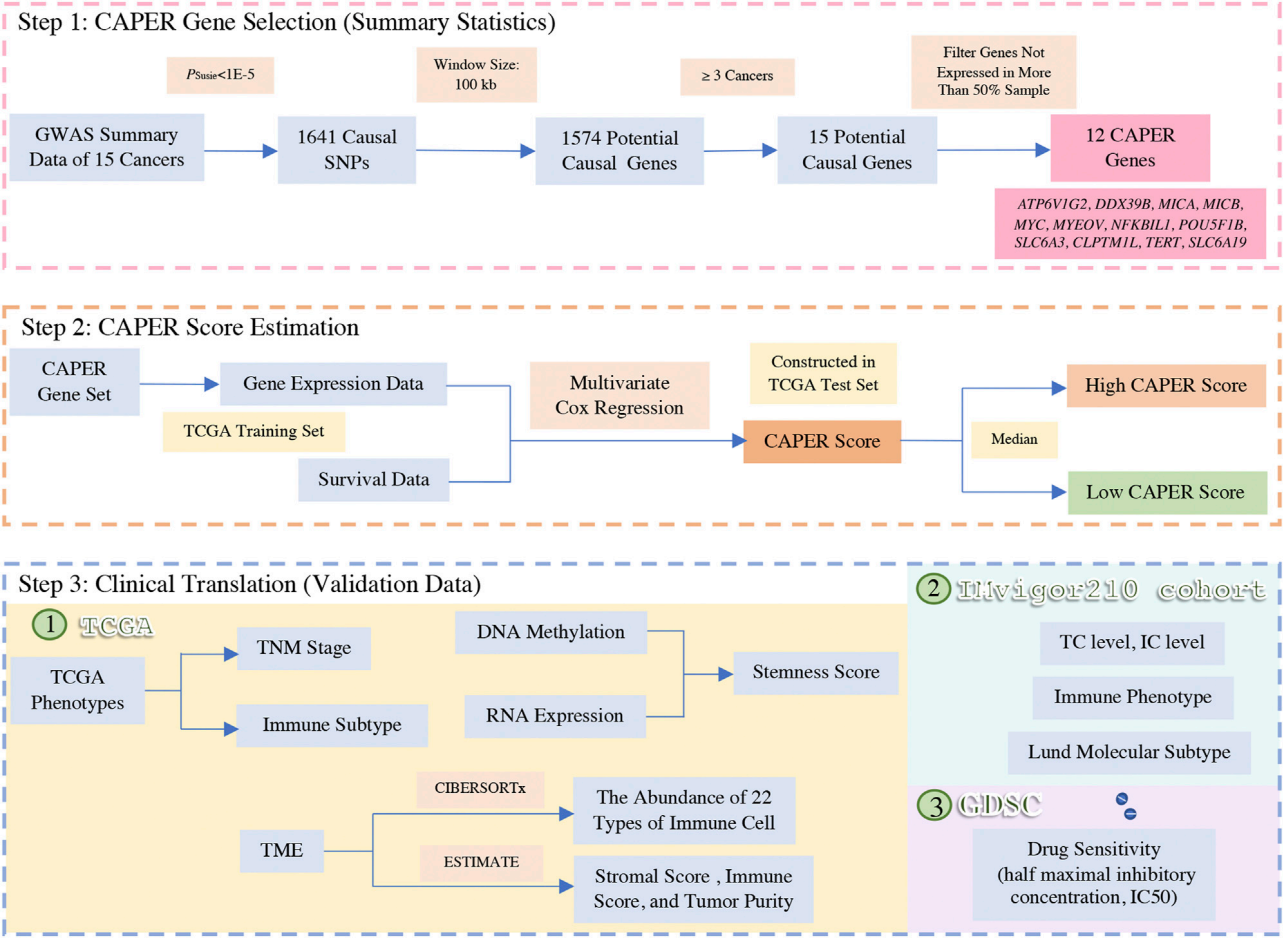
Workflow for CAPER score construction and its clinical translation.

In this study, we developed a novel strategy to define causal genes with the assumption of pan-cancer, to construct the CAusal Pan-cancER gene (CAPER) score, and to validate its performance using internal and external validation (Figure 1). Based on the workflow, we identified causal genes related to multiple cancers, used the same gene panel to differentiate cancer patients, and validated the efficiency of the gene panel in external validation sets (Li et al., 2021).

## Materials and Methods

### Genome-Wide Associate Studies Data Process

We obtained 15 GWAS summary statistics of common cancers, including bladder cancer (BLCA, Prev. = 23.0/100,000), breast cancer (BRCA, Prev. = 125.2/100,000), cervical squamous cell carcinoma and endocervical adenocarcinoma (CESC, Prev. = 12.4/100,000), colorectal cancer (COADREAD, Prev. = 55.9/100,000), esophageal or stomach adenocarcinoma (ESCASTAD, Prev. = 15.1/100,000), kidney cancer (KC, Prev. = 14.5/100,000), lung cancer (LC, Prev. = 35.7/100,000), lymphocytic leukemia (LL, Prev. = 10.1/100,000), melanoma (MM, Prev. = 18.3/100,000), oral cavity and pharyngeal cancer (OCPC, Prev. = 3.3/100,000), ovarian cancer (OV, Prev. = 13.5/100,000), pancreatic adenocarcinoma (PAAD, Prev. = 7.9/100,000), prostate cancer (PRAD, Prev. = 120.1/100,000), thyroid carcinoma (THCA, Prev. = 10.2/100,000), and uterine corpus endometrial cancer (UCEC, Prev. = 29.4/100,000) (Li et al., 2012; Leo et al., 2017; Phelan et al., 2017; O’Mara et al., 2018; Schumacher et al., 2018; Zhou et al., 2018; Rashkin et al., 2020). The average number of SNPs was 1,144,365 (median = 1,293,959, ranging from 269,795 to 1,298,901) and the average sample size was 273,861 (median = 399,215, ranging from 7,785 to 417,127). In addition, we used Linkage Disequilibrium SCore regression (LDSC) (Bulik-Sullivan et al., 2015) to estimate the observed and liability heritability of each cancer and the genetic correlation between each pair of cancer types. We identified 28 significant pairs of cancer types (*p* < 0.05), among which OV and UCEC presented the lowest *p-*value (*p* = 3.69E-7) (Supplementary Figure S1). Since all the GWASs had been performed on patients with European ancestry, we used the LD scores of the European population of the 1000 Genome Project (1 KGP) to estimate the heritability of each cancer (Consortium, 2015). Details on the 15 summary statistics are provided in Table 1.

**TABLE 1:** Summary of GWAS summary statistics in 15 types of cancer.

Cancer type	No. SNP	$h^{2}$	Samples size	Prev. (/100,000)
BLCA	1,293,985	0.08	412,592	23
BRCA	1,016,724	0.14	194,153	125.2
CESC	269,795	0.36	9,347	12.4
COADREAD	1,298,901	0.23	387,318	55.9
ESCASTAD	1,293,959	0.14	411,441	15.1
KC	1,293,994	0.09	411,688	14.5
LC	1,293,976	0.15	412,835	35.7
LL	1,293,985	0.14	411,202	10.1
MM	1,293,929	0.08	417,127	18.3
OCPC	1,293,988	0.04	411,573	3.3
OV	1,227,160	0.0042	85,426	13.5
PAAD	518,381	0.06	7,785	7.9
PRAD	1,202,176	0.16	140,254	120.1
THCA	1,293,992	0.21	411,112	10.2
UCEC	1,280,529	0.03	121,885	29.4

$h^{2}$ : Heritability estimated.

Prev. (/100,000): Estimated number of prevalent cases in 2020 (proportions per 100,000).

BLCA: bladder cancer, BRCA: breast cancer, CESC: cervical squamous cell carcinoma and endocervical adenocarcinoma, COADREAD: colorectal cancer, ESCASTAD: esophageal or stomach adenocarcinoma, KC: kidney cancer, LC: lung cancer, LL: lymphocytic leukemia, MM: melanoma, OCPC: oral cavity and pharyngeal cancer, OV: ovarian cancer, PAAD: pancreatic adenocarcinoma, PRAD: prostate cancer, THCA: thyroid carcinoma, and UCEC: uterine corpus endometrial cancer.

Furthermore, we used the GENCODE database (v25) to map the SNPs to gene positions. In total, we retained 19,201 protein-coding genes selected from 60,252 transcripts in autosomes. Then, the SNPs located 100 kb upstream and downstream of a specific gene were selected to perform fine-mapping. After mapping to the gene and intersecting using the reference panel, 1,403,668 SNPs remained. We used PLINK (v1.9b6.22) to estimate the LD matrix of each gene.

### Potential Causal Gene Set Identification

We used SuSiE, a fine-mapping method, to identify the causal SNPs in the 15 GWAS summary datasets with the aid of the *susieR* package (v0.11.42) (Wang et al., 2020a) in R software. We also used 1 KGP EUR samples as the LD reference panel. According to SuSiE manual, we set the maximum number of causal variants in the region to 10. Based on the results of previous studies (Fadista et al., 2016; Schaid et al., 2018), we set the significant level to 1E-5. A specific gene with causal SNPs was defined as a potential causal gene. We repeated the fine-mapping procedure for each type of cancer. Finally, the potential causal gene set consisted of genes regarded as casual genes of at least three types of cancers.

We also used the Molecular Signatures Database (MSD) to evaluate the overlapping of our candidate genes with regard to common processes, pathways, and underlying biological themes, while considering an FDR *q*-value of less than 0.05 and a minimum gene set size of two as statistically significant (Subramanian et al., 2005; Liberzon et al., 2011; Liberzon et al., 2015).

### The Cancer Genome Atlas Data Process

To verify the clinical translation of the potential causal gene set, we downloaded two types of TCGA data: (1) molecular data, which included gene expression (HTSeq-FPKM) (log2(FPKM+1)) and DNA methylation data; and (2) clinical data, which included age, sex, and survival time. These data were downloaded from the University of California Santa Cruz (UCSC) Xena browser (Goldman et al., 2018). We used data on all 11,057 samples and 60,483 transcripts available on 33 different types of cancer. We transformed the Ensembl IDs to symbols, using the *biomaRt* package (v.2.46.3) (Durinck et al., 2009). Specifically, we used the average level to represent the gene with multiple Ensembl ID mapping with a single symbol. We obtained the sum of the genes from the 33 datasets and calculated the average expression level for genes that contained more than one transcript. We also filtered out the samples that were (1) non-European ancestry and (2) missing tumor stage data. Genes that were not expressed (FPKM = 0) in more than 50% of samples were excluded from the expression data of our causal gene set. After quality control, we obtained 38,596 genes, including 12 CAPER genes, and 4,842 individuals with 21 different types of cancer.

### CAusal Pan-cancER Gene Score Estimation

First, we divided the TCGA sample into two parts: the training set (80%), used to construct the CAPER score, and the test set (20%), used to perform internal validation. Of the 4,842 individuals, 3,873 were allocated to the training set and 969 were allocated to the test set (Supplementary Table S1). The 21 types of cancer included were adrenocortical carcinoma (ACC), BLCA, BRCA, cholangiocarcinoma (CHOL), colon adenocarcinoma (COAD), esophageal carcinoma (ESCA), head and neck squamous cell carcinoma (HNSC), kidney chromophobe (KICH), kidney renal clear cell carcinoma (KIRC), kidney renal papillary cell carcinoma (KIRP), liver hepatocellular carcinoma (LIHC), lung adenocarcinoma (LUAD), lung squamous cell carcinoma (LUSC), mesothelioma (MESO), PAAD, rectum adenocarcinoma (READ), skin cutaneous melanoma (SKCM), stomach adenocarcinoma (STAD), testicular germ cell tumors (TGCT), THCA, and uveal melanoma (UVM). The training set was used to perform Cox regression on each gene after adjusting for age, sex, and tumor stage. We used the *survival* package (v3.2–11) to fit the Cox regression data, and the *survminer* package (v0.4.9) was used to perform survival analysis and visualization. We obtained the association between each gene and the disease-specific overall survival rate.

Second, we used a causal gene set to fit the multivariable Cox regression adjusted for age, sex, and tumor stage. The model used was as follows:

$$CAPER\;Score={\hat{\beta }}_{CAPER}X_{CAPER}$$ (1)

where ${\hat{\beta }}_{CAPER}$ was a vector of the coefficients of CAPER genes obtained from the multivariable regression and $X_{CAPER}$ was a matrix of expression levels of CAPER genes. Using Eq. 1, we constructed the CAPER score for each individual in the test set and regarded the 12 potential causal genes identified as CAPER genes. Using the median CAPER score, we classified the individuals into two groups: (1) the high-CAPER group and (2) the low-CAPER group.

In addition, we used the TCGA test set to perform three types of sensitivity analyses. (1) To show the performance of the CAPER score for a shorter survival time, we selected all samples with survival <5 years. (2) To show the performance of the CAPER score in a smaller sample size, we randomly selected 80 samples. (3) To show the effectiveness and accuracy of the CAPER score, we randomly selected 12 genes, namely, *DGFRL, GLRX5, KCNJ14, SMARCAL1, FTH1P16, CDK5, WDFY1, TMEM266, RAD21, NAA16, AGPS,* and *FBXO39*, to construct the random CAPER score. We used the log-rank test for all three analyses.

### Clinical Translation

We performed a series of analyses to investigate the clinical translation of the CAPER score, using the TCGA test set, IMvigor210 cohort, and Genomics of Drug Sensitivity in Cancer (GDSC) (Yang et al., 2012; Tomczak et al., 2015; Mariathasan et al., 2018).

First, using the TCGA test set, we defined the association between CAPER score and TNM staging, tumor histological grade, and vascular tumor cell types. Specifically, the pathological stages of the primary tumor (T) were divided into two groups: (1) Tis (tumor *in situ*) and T1, and (2) T2 or larger. The staging of distant metastasis (M) is defined as M0 and M1. In addition, we detected the association between the CAPER score and immune subtypes, including C1 (wound healing), C2 (IFN-γ dominant), C3 (inflammatory), C4 (lymphocyte depleted), C5 (immunologically quiet), and C6 (TGF-β dominant), which were filtered because the sample size was below 40. These immune subtypes were proven to be associated with prognosis, genetic, and immune-modulatory alterations. These factors may shape the specific types of immune environments that we observed and indicate response to therapy or prognosis (Thorsson et al., 2018). We also showed the association between the high-CAPER group and the low-CAPER group, as well as the immune subtype of each gene.

Second, we used the TCGA test set to define the association between the CAPER score and the tumor microenvironment. On the one hand, we estimated the immune score, stromal score, and tumor purity using the *limma* (v3.46.0) (Ritchie et al., 2015) and the *estimate* (v1.0.13) packages (Yoshihara et al., 2013). ESTIMATE is a method that uses gene expression signatures to infer the fraction of stromal and immune cells in the tumor samples. We estimated the correlation between the CAPER score, the three metrics, and identified differences between the two scores and tumor purity of the high- and low-CAPER group. After filtering out data on 3 cancer types (CHOL, KICH, and UVM) with a sample size below 10 (low sample size cancers), we also estimated the Spearman correlation between single CAPER gene expression and three metrics in each cancer. On the other hand, we used the support vector regression (SVR) on CIBERSORTx (Steen et al., 2020) to deconvolve RNA admixtures to the abundance of 22 types of immune cells in TCGA samples to further observe the tumor microenvironment (TME) (Chen et al., 2018). Following the CIBERSORTx manual, we set the number of permutations to 100. We uploaded RNA-seq FPKM data and set quantile normalization to discern the recommended setting (Craven et al., 2021). We filtered out immune cell types with an average proportion lower than 2%, and 14 types of immune cells were included into the final analysis. We identified differences in the immune cell abundance between the high- and low-CAPER groups. After filtering out 3 types of cancers with a small sample size, we estimated the Spearman correlation between the CAPER score and the abundance of 14 immune cells in 18 types of cancers.

Third, we used the stemness score based on DNA methylation (DNAss) and RNA expression (RNAss) obtained from UCSC. The stemness score is defined as the quantification of stemness and is associated with tumor progression, therapeutic resistance, and recurrence. DNAss indicates epigenetic features while RNAss indicates gene expression (Malta et al., 2018; Pei et al., 2020). We estimated the Spearman correlation between single CAPER gene expression and the stemness score in 18 types of cancer. For the CAPER score, we estimated the Spearman correlation between the CAPER score and the stemness score and conducted a Wilcoxon rank-sum test to compare the statistical significance of the stemness score between high- and low-CAPER groups.

Fourth, we used the IMvigor210 cohort to verify the robustness and efficiency of the CAPER score. Data were downloaded using the Imvigor210CoreBiologies package (v1.0.0). The cohort data included immune phenotypes (immune inflamed, immune excluded, and immune desert), Lund molecular subtypes, IC-Level (level of immunohistochemistry-assessed PD-L1 staining on immune cells), and TC-Level (level of immunohistochemistry-assessed PD-L1 staining on tumor cells). We used the coefficient estimated using the training set in TCGA to construct the CAPER score for the Imvigor210 cohort and investigated the difference in variables in the high- and low-CAPER groups.

Finally, using GDSC, the largest free public database of information on drug sensitivity in cancer cells and molecular markers of drug response (Yang et al., 2012), we identified the association between the CAPER score and drug sensitivity. We constructed the CAPER score for each cell line sample and conducted a Spearman correlation analysis between the CAPER score and IC50 value, the half maximal inhibitory concentration, which is an established measurement of drug efficacy (Aykul and Martinez-Hackert, 2016).

## Results

### Identification of Potential Causal Genes

**FIGURE 2:**
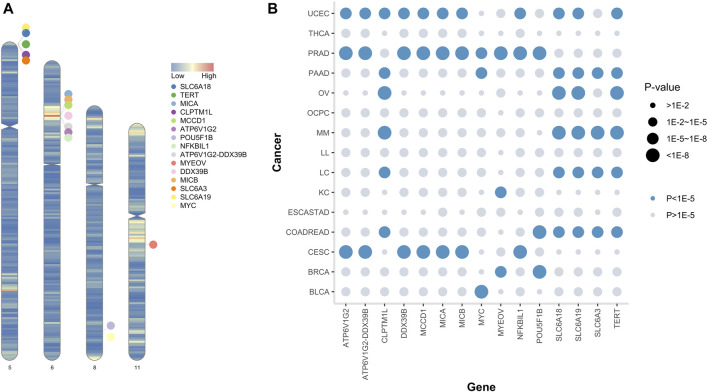
Summary of the CAPER genes. **(A)** Ideogram of the 15 CAPER genes (the color of each chromosome indicates gene density across the human genome). **(B)** The bubble plot shows the minimum *p*-value of each causal gene in each cancer dataset.

Using SuSiE (Wang et al., 2020a), we identified 54 causal SNPs in 15 genes (*ATP6V1G2, ATP6V1G2-DDX39B, CLPTM1L, DDX39B, MCCD1, MICA, MICB, MYC, MYEOV, NFKBIL1, POU5F1B, SLC6A18, SLC6A19, SLC6A3*, and *TERT*). Detailed information of the causal SNPs and potential causal genes is shown in Table 2 and Figure 2, and Supplementary Table S2. MDB showed that 13 were significantly enriched in breast tumor, hepatocellular carcinoma, substance transport-related, and certain other pathways, while 12 were located in the cytogenic region of 6p21 or 5p15 (FDR *q*-value < 0.05, Supplementary Table S3).

**TABLE 2:** Summary of the 15 potential causal genes.

Gene	CHR	Start	End	*p* (min)	Cancer	No. SNP
*SLC6A19*	5	1,201,710	1,225,232	1.39E-12	MM, OV, LC, PAAD, UCEC	13
*SLC6A18*	5	1,225,470	1,246,304	5.32E-13	MM, OV, LC, PAAD, UCEC	23
*TERT*	5	1,253,262	1,295,184	5.32E-13	MM, OV, LC, PAAD, UCEC	23
*CLPTM1L*	5	1,317,859	1,345,214	5.32E-13	MM, OV, LC, PAAD, UCEC	23
*SLC6A3*	5	1,392,905	1,445,545	5.32E-13	MM, LC, PAAD	21
*MICA*	6	31,367,561	31,383,092	2.30E-11	CESC, PRAD, UCEC	6
*MICB*	6	31,462,658	31,478,901	2.30E-11	CESC, PRAD, UCEC	7
*MCCD1*	6	31,496,494	31,498,009	2.30E-11	CESC, PRAD, UCEC	10
*DDX39B*	6	31,497,996	31,510,225	2.30E-11	CESC, PRAD, UCEC	12
*ATP6V1G2-DDX39B*	6	31,497,996	31,514,385	2.30E-11	CESC, PRAD, UCEC	12
*ATP6V1G2*	6	31,512,239	31,516,204	2.30E-11	CESC, PRAD, UCEC	12
*NFKBIL1*	6	31,514,647	31,526,606	2.30E-11	CESC, PRAD, UCEC	12
*MYC*	8	128,747,680	128,753,680	1.77E-09	BLCA, PRAD, PAAD	4
*POU5F1B*	8	128,426,535	128,432,314	5.73E-186	PRAD, BRCA, COADREAD	11
*MYEOV*	11	69,061,605	69,182,494	3.84E-97	PRAD, BRCA, KC	11

### Construction of the Causal Pan-cancER Gene Score

**FIGURE 3:**
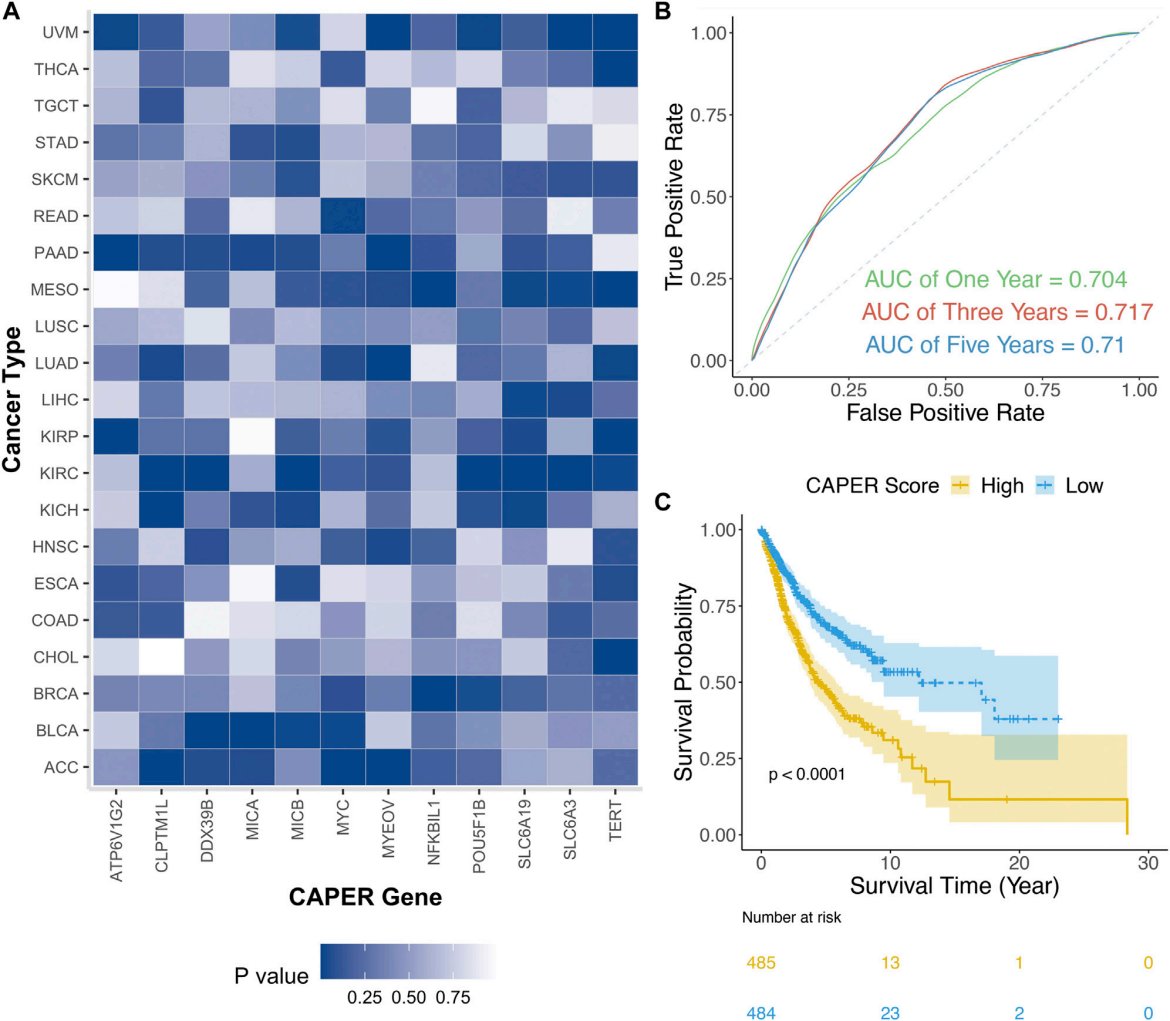
Summary of the CAPER score. **(A)** Heatmap showing the *p*-value of each CAPER gene in the univariate Cox regression performed on each type of cancer. **(B)** ROC of the multivariate Cox regression conducted on the TCGA test set. We estimated the AUC of 1-year (green), 3-year (Red), and 5-year (Blue) survival. **(C)** K-M curve of the high- and low-CAPER score groups. The HR of the CAPER score was statistically significant to the survival time with a *p*-value of <0.001 and was adjusted for age, sex, and tumor stage.

The training data of TCGA was used to select 12 CAPER genes, investigate their pan-cancer association, and construct the CAPER score. Through univariate Cox regression, we defined 6 significant causal genes with an average *p*-value of 0.01 (median = 1.87E-06, ranging from 4.62E-09 to 0.04) by adjusting for age, sex, and tumor size. For example, from among the 12 CAPER genes, *MYEOV* was the gene with the greatest risk (HR = 1.09, 95% CI: 1.06–1.13), while *ATP6V1G2* was the gene that offered the largest protection effect (HR = 0.68, 95% CI: 0.59–0.78). Details of the univariate analysis are presented in Table 3 and Figure 3A.

**TABLE 3:** Summary of the univariable Cox regression analysis conducted on 12 CAPER genes in the TCGA training set *.

Gene	Coef.	SE (coef.)	*Z*	*p*	HR (95%CI)
*ATP6V1G2*	−0.38	0.07	−5.57	2.59E-8	0.68 (0.59, 0.78)
*DDX39B*	0.00	0.03	0.06	0.96	1.00 (0.95, 1.06)
*MICA*	0.03	0.04	0.87	0.39	1.03 (0.96, 1.11)
*MICB*	0.06	0.03	2.01	0.045	1.06 (1.00, 1.12)
*MYC*	0.09	0.02	4.77	1.87E-6	1.09 (1.05, 1.13)
*MYEOV*	0.09	0.02	5.86	4.62E-9	1.09 (1.06, 0.78)
*NFKBIL1*	−0.07	0.04	−1.81	0.070	0.93 (0.87, 1.01)
*POU5F1B*	−0.36	0.08	−4.57	4.81E-6	0.70 (0.60, 0.82)
*SLC6A3*	−0.03	0.02	−1.85	0.065	0.97 (0.94, 1.00)
*CLPTM1L*	0.07	0.04	1.67	0.095	1.07 (0.99, 1.16)
*TERT*	0.03	0.05	0.62	0.53	1.03 (0.93, 1.15)
*SLC6A19*	−0.07	0.03	−2.75	6.03E-3	0.93 (0.89, 0.98)

* The effect sizes of genes are adjusted by age, sex, and tumor stage.

Furthermore, we performed a multivariate Cox regression to construct the CAPER score using the TCGA training set (Table 4). We estimated the time-dependent ROC curves for multivariate Cox regression for 1-, 3-, and 5-year survival (AUC = 0.704, 0.717, and 0.710, respectively) using TCGA test data (Figure 3B). The time-dependent ROC curves in the TCGA training set are provided in Supplementary Figure S2. The formula used to construct the CAPER score is as follows:

$$\begin{array}{l} CAPER\;score\;=-0.308*ATP6V1G2+0.058*DDX39B\\ +0.044*MICA-0.040*MICB\\ +0.083*MYC+0.076*MYEOV\\ -0.077*NFKBIL1-0.459*POU5F1B\\ -0.021*SLC6A3\;+\;0.074*CLPTM1L\\ +\;0.018*TERT-0.064*SLC6A19 \end{array}$$ (2)

**TABLE 4:** Summary of the multivariable Cox regression conducted on 12 CAPER genes in the TCGA training set *.

Gene	Coef	SE (coef.)	*Z*	*p*	HR (95%CI)
*ATP6V1G2*	−0.28	0.08	−3.44	5.79E-0	0.75 (0.64, 0.89)
*DDX39B*	0.06	0.04	1.67	0.095	1.06 (0.99, 1.14)
*MICA*	0.06	0.05	1.23	0.22	1.06 (0.97, 1.15)
*MICB*	−0.03	0.04	−0.89	0.37	0.97 (0.90, 1.04)
*MYC*	0.12	0.02	5.20	1.97E-7	1.13 (1.08, 1.18)
*MYEOV*	0.08	0.02	4.14	3.45E-5	1.08 (1.04, 1.12)
*NFKBIL1*	−0.09	0.05	−1.88	0.060	0.92 (0.84, 1.00)
*POU5F1B*	−0.41	0.08	−4.87	1.11E-6	0.66 (0.56, 0.78)
*SLC6A3*	−0.02	0.02	−0.72	0.47	0.98 (0.94, 1.03)
*CLPTM1L*	0.06	0.05	1.32	0.19	1.07 (0.97, 1.17)
*TERT*	0.02	0.07	0.23	0.82	1.02 (0.89, 1.16)
*SLC6A19*	−0.06	0.03	−1.80	0.071	0.94 (0.88, 1.01)

*The effect sizes of genes are adjusted by age, sex, and tumor stage.

The HR for the CAPER score is 3.75 (95% CI: 2.65–5.32). In addition, Kaplan–Meier curves were used to detect differences in survival between the high- and low-CAPER groups, which was found to be significant (log-rank test, *p* < 0.0001), with the median survival time of the high-CAPER group being shorter (Figure 3C).

In addition, sensitivity analyses indicated the effective construction procedure and accurate prognosis performance. When we used 5 years as the cutoff, the difference in survival between high- and low-CAPER groups was found to be significant (log-rank test; *p* < 0.0001) (Supplementary Figure S3A). When a small sample size was used (i.e., 80 individuals), the difference in survival between the high- and low-CAPER groups was also significant (log-rank test; *p* = 0.011) (Supplementary Figure S3B). A random gene set was used, and although three genes showed significant differences, the difference between high- and low-CAPER groups was not significant (log-rank test, *p* = 0.54) (Supplementary Figure S4 and Supplementary Table S4).

### Internal Validation of the CAusal Pan-cancER Gene Score Using The Cancer Genome Atlas Test Set

TCGA test data were used to explore the clinical translation of the CAPER score and its potential application for therapeutic and prognostic purposes in cancer management. We used the CAPER score to classify patients into high- and low-CAPER groups and tested differences in clinical metrics, TME, and the stemness score between the two groups.

**FIGURE 4:**
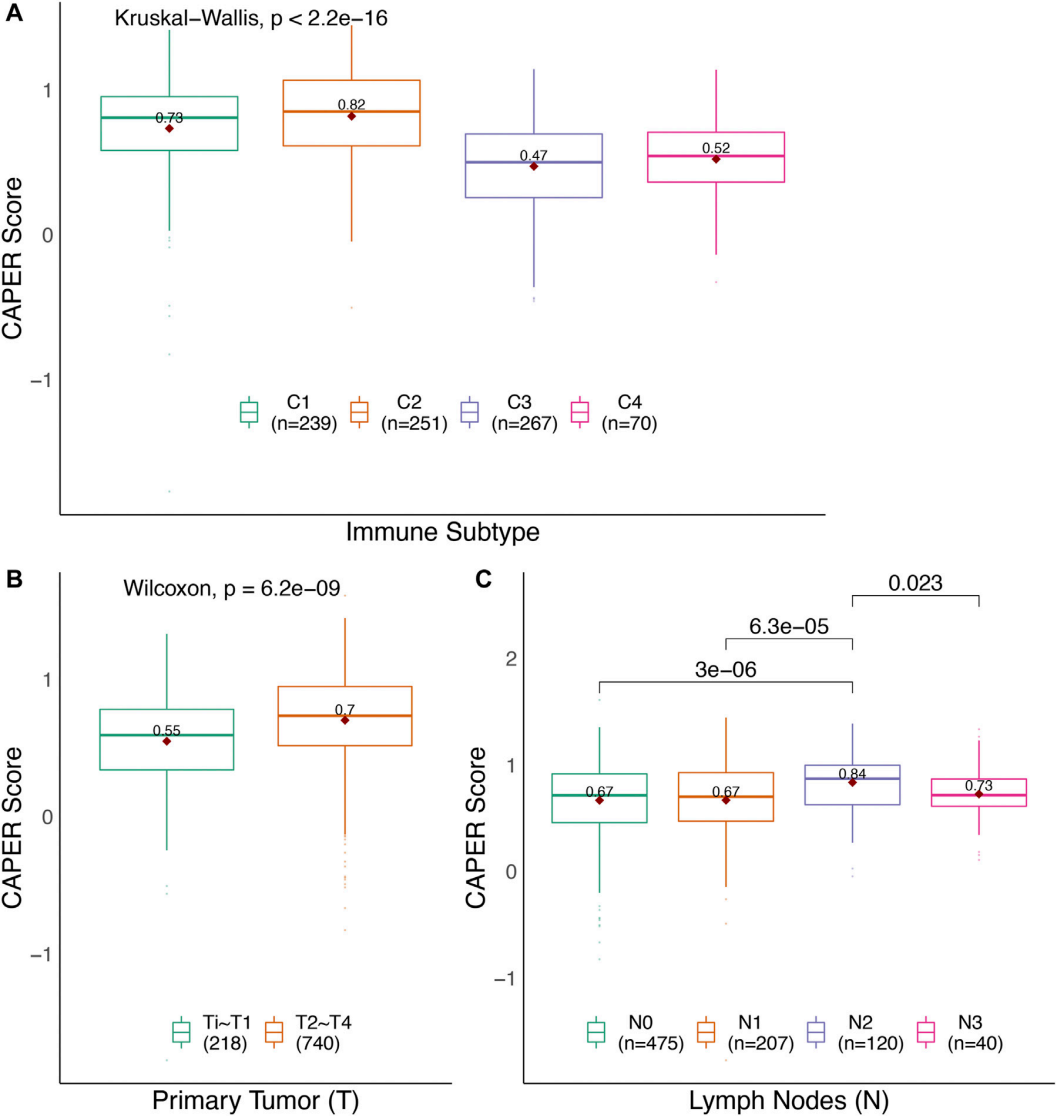
Summary of the differences between the three tumor-related variables in the different CAPER groups using the TCGA test set. **(A)** immune subtypes; **(B)** the pathological stage of the primary tumor (T); **(C)** the pathological stage of the lymph nodes (N).

**FIGURE 5:**
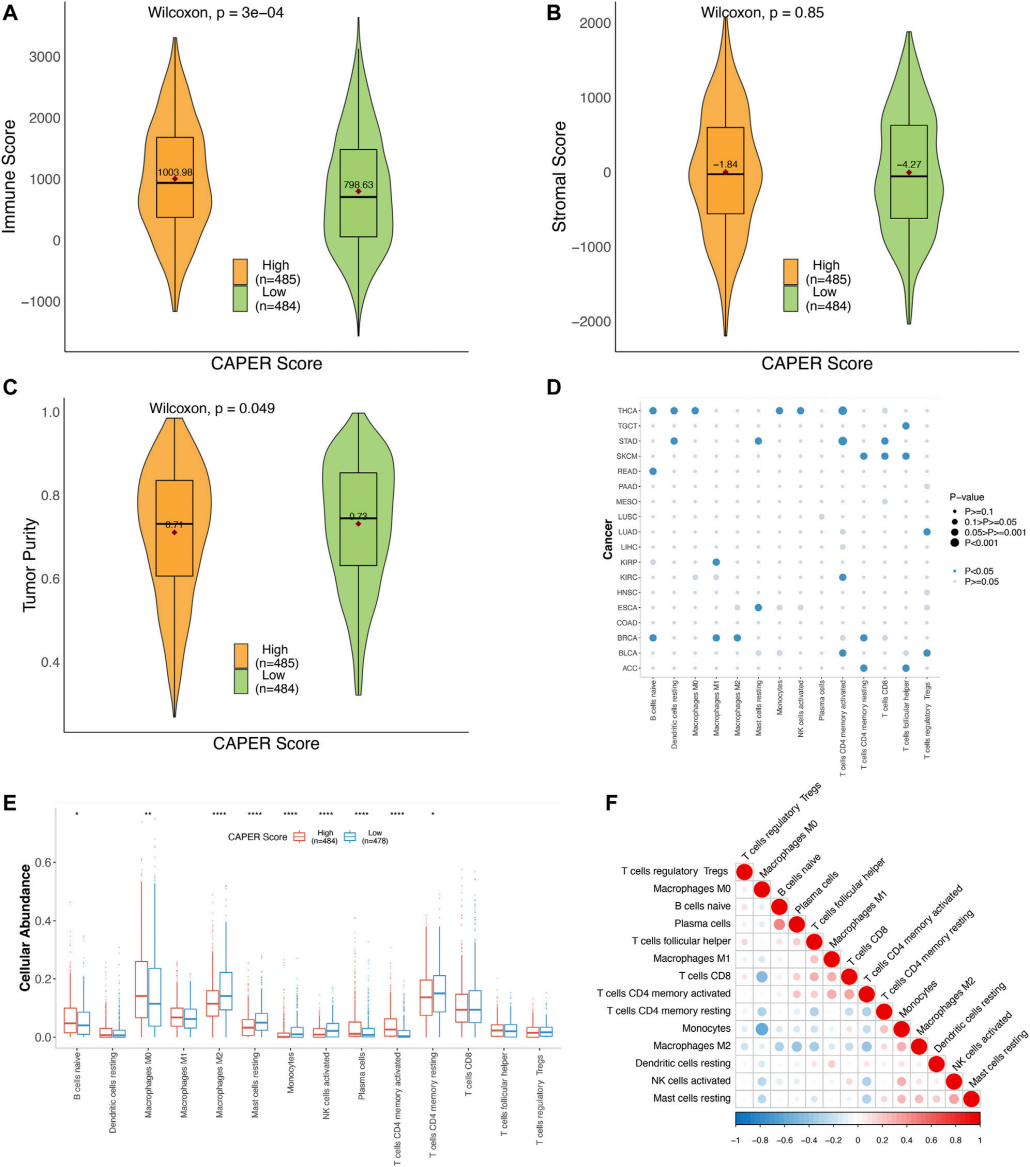
Summary of the correlation between TME and CAPER score or CAPER gene expression using the TCGA test set. **(A)** Differences in the immune score between the CAPER score groups. **(B)** Differences in the stromal score between the CAPER score groups. **(C)** Differences in tumor purity between the CAPER score groups. **(D)** The bubble plot shows the *p*-value of Spearman correlation tests conducted on 18 types of cancer. **(E)** The boxplot cellular abundance differential score between the high- and low-CAPER score groups (*p < 0.05, **p < 0.01, and ***p < 0.001). **(F)** The autocorrelation plot of cellular abundance.

First, we analyzed the association between CAPER score and immune subtypes and TNM stage (Figure 4 and Table 5). The Kruskal–Wallis test found the difference in each immune subtype to be significant ( $\chi^{2}=164.21$ , *p* < 2.2E-16) with an average CAPER score of 0.73, 0.82, 0.47, and 0.52, respectively. As expected, the frequencies of each immune subtype were different between the high- and low-CAPER groups ( $\chi^{2}=130.05$ , *p* < 2.2E-16). Then, the average CAPER score in the different primary tumors (T stage) in the high- and low-CAPER groups were 0.55 and 0.7. Again, the Kruskal–Wallis test showed that the difference in each subtype was significant (*p* = 6.22E-09). As expected, the frequencies of each primary subtype were different between the high- and low-CAPER groups ( $\chi^{2}=20.35$ , *p* = 6.44E-06). The average CAPER score in the different primary tumors (N stage) was 0.67, 0.67, 0.84, and 0.73, respectively. The Kruskal–Wallis test showed that the difference in each subtype was significant ( $\chi^{2}=22.94$ , *p* = 4.15E-05). As expected, a significant difference in the frequencies of each subtype was found between the groups ( $\chi^{2}=17.68$ , *p* = 5.12E-04). The average CAPER score for distant metastasis (M stage) was 0.66 and 0.70 for M0 and M1, respectively. The Wilcoxon rank-sum test showed that the difference in each subtype was non-significant (*p* = 0.40). The association between M stage and CAPER groups also shows no statistical significance ( $\chi^{2}=0.40$ , *p* = 0.53). Second, we analyzed the association between the CAPER score and TME (Figure 5 and Supplementary Figure S5). We estimated the immune score, stromal score, and tumor purity. The immune score was positively related to the CAPER score (Spearman correlation test, *ρ* = 0.15, *p* = 3.02E-06), while the low-CAPER group had a lower immune score than the high-CAPER group (Wilcoxon rank-sum test, *p* = 3.02E-04). Consistent with the results of the survival analysis, patients with a low CAPER score may have fewer immune cells. The association between stromal score and CAPER score between the high- and low-CAPER groups showed no statistical significance (Spearman correlation test, *ρ* = 0.0059, *p* = 0.8543, Wilcoxon rank-sum test, *p* = 0.8506). The tumor purity was negatively related to the CAPER score (Spearman correlation test, *ρ* = −0.08, *p* = 0.0107). The low-CAPER group showed higher tumor purity than the high-CAPER group (Wilcoxon rank-sum test, *p* = 0.0492). On the other hand, we estimated the cellular abundance of 22 types of immune cells obtained from CIBERSORTx and filtered immune cell types with an average proportion that was lower than 2%. We selected 14 types of immune cells and explored their associations with the CAPER score (Supplementary Figure S6). Consistent with the results of the survival analysis and ESTIMATE, we found that infiltration of naïve B cells (Wilcoxon rank-sum test, *p* = 0.027), macrophages M0 (*p* = 0.001), plasma cells (*p* = 1.93E-05), and CD4 memory activated T cells (*p* = 3.49E-22) was higher in the high-CAPER group, while the infiltration of M2 macrophages (*p* = 1.24E-08), resting mast cells (*p* = 5.27E-11), monocytes (*p* = 5.92E-10), activated NK cells (*p* = 1.72E-10), and CD4 memory resting T cells (*p* = 0.035) was higher in the low-CAPER group.

**TABLE 5:** Summary of the association between clinical categorical variables and the CAPER score in TCGA.

Variables	High-CAPER *n* (%)	Low-CAPER *n* (%)	*χ* ^2^	*p*
The Primary Tumor				
Ti, T1	79 (16.5%)	139 (29.0%)	20.35	6.44E-6
T2, T3, T4	399 (83.5%)	341 (71.0%)		
The Lymph Nodes				
N0	246 (53.7%)	229 (59.6%)	17.68	5.12E-4
N1	103 (22.5%)	104 (27.1%)		
N2	86 (18.8%)	34 (8.9%)		
N3	23 (5.0%)	17 (4.4%)		
Metastasis				
M0	332 (93.0%)	354 (94.4%)	0.40	0.53
M1	25 (7.0%)	21 (5.6%)		
Immune Subtypes				
C1	150 (37.2%)	89 (21.0%)	130.05	<2.2E-16
C2	169 (41.9%)	82 (19.3%)		
C3	66 (16.4%)	201 (47.4%)		
C4	18 (4.5%)	52 (12.3%)		

Finally, we analyzed the correlation between the CAPER score and the stemness score constructed using gene expression and DNA methylation data (Supplementary Figure S7). The RNA stemness score (Spearman correlation test, *ρ* = 0.29, *p* < 2.2E-16) and the DNA stemness score were both positively correlated to the CAPER score (Spearman correlation test, *ρ* = 0.32, *p* < 2.2E-16). The high-CAPER group had a higher DNA (Wilcoxon rank-sum test, *p* = 6.9E-15) and RNA (Wilcoxon rank-sum test, *p* = 1.5E-13) stemness score, indicating stronger tumor stem cell activity and a lower degree of tumor differentiation.

### External Validation of the CAusal Pan-cancER Gene Score Using IMvigor210

**FIGURE 6:**
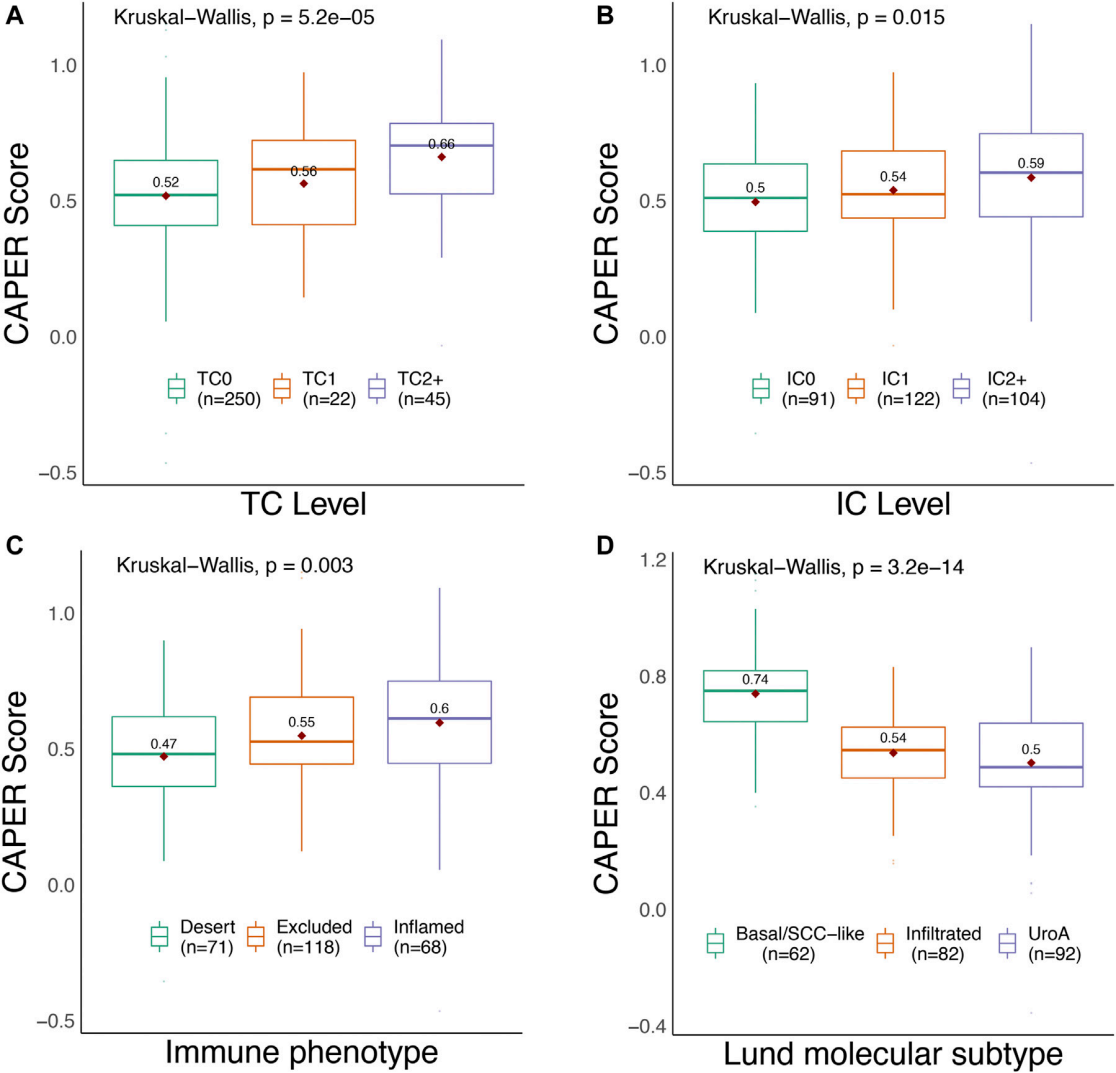
Summary of the differences between the four tumor phenotypes in different CAPER groups using IMvigor210. **(A)** TC levels; **(B)** IC levels; **(C)** immune phenotypes; **(D)** Lund molecular subtypes.

The IMvigor210 cohort was analyzed to verify the robustness and efficiency of the CAPER score; we observed that the CAPER score was significantly associated with the three types of clinical metrics in IMvigor210 (Table 6 and Figure 6). For the clinical phenotypes, differences in the CAPER score between patients with different TC levels were statistically significant (Kruskal–Wallis test, *p* = 5.2E-05). The CAPER score of TC2+ patients was higher than that of TC0 patients (Wilcoxon rank-sum test, *p* = 1.1E-05). The differences in CAPER score between patients with different IC levels were statistically significant (Kruskal–Wallis test, *p* = 0.015). The CAPER score of IC2+ patients was higher than that in IC0 patients (Wilcoxon rank-sum test, *p* = 3.9E-03). In addition, we analyzed CAPER score differences between immune phenotypes using IMvigor210. The differences in CAPER score between patients with different immune phenotypes were statistically significant (Kruskal–Wallis test, *p* = 0.003). We observed that the “immune inflamed” type had a higher CAPER score than the “desert” type (Wilcoxon rank-sum test, *p* = 1.2E-03). The difference in Lund molecular subtypes was significant in different CAPER groups (Kruskal–Wallis test, *p* = 3.2E-14). The CAPER score of the “Basal/SCC-like” type was higher than that of the “Infiltrated” (Wilcoxon rank-sum test, *p* = 1.5E-11), and “UroA” (Wilcoxon rank-sum test, *p* = 7.2E-13) types. As expected, differences between the high- and low-CAPER groups were also significant for the three tumor immunity-related variables indicated above (TC levels, *p* = 2.53E-03, immune subtypes, *p* = 0.01, and Lund molecular subtypes, *p* = 7.28E-09, respectively).

**TABLE 6:** Summary of the association between four tumor immunity-related variables and CAPER score in the IMvigor210 cohort.

Variables	High-CAPER *n* (%)	Low-CAPER *n* (%)	*χ* ^2^	*p*
IC Levels				
IC0	41 (25.8%)	50 (31.6%)	5.55	0.06
IC1	56 (35.2%)	66 (41.8%)		
IC2+	62 (39.0%)	42 (26.6%)		
TC Levels				
TC0	113 (71.1%)	137 (86.7%)	11.96	2.53E-3
TC1	14 (8.8%)	8 (5.1%)		
TC2+	32 (20.1%)	13 (8.2%)		
Immune Phenotypes				
Desert	27 (21.6%)	44 (33.3%)	9.19	0.01
Excluded	55 (44.0%)	63 (47.7%)		
Inflamed	43 (34.4%)	25 (18.9%)		
Lund Molecular Subtype				
UroA	37 (27.8%)	55 (53.4%)	37.48	7.28E-9
Infiltrated	41 (30.8%)	41 (39.8%)		
Basal/SCC-like	55 (41.4%)	7 (6.8%)		

### Potential Therapeutic Value of the CAusal Pan-cancER Gene Score

We analyzed the correlation between the CAPER score and drug sensitivity in the GDSC using Spearman correlation and identified 81 significantly correlated drugs between the CAPER score and drug sensitivity (Supplementary Table S5). For example, the CAPER score was negatively correlated with the sensitivity of Afatinib (*ρ* = −0.28, *p* = 6.71E-17), Trametinib (*ρ* = −0.23, *p* = 2.12E-12), Selumetinib (*ρ* = −0.22, *p* = 1.36E-11), and Refametinib (*ρ* = −0.21, *p* = 2.22E-10), which indicated a lower CAPER score against their higher level of drug sensitivity. The CAPER score was positively correlated to the sensitivity of Axitinib (*ρ* = 0.14, *p* = 2.56E-05), SB52334 (*ρ* = 0.13, *p* = 5.61E-05), GSK269962A (*ρ* = 0.12, *p* = 4.2E-04), and Quizartinib (*ρ* = 0.10, *p* = 3.0E-03). Furthermore, we found that drugs with sensitivity that was negatively correlated with the CAPER score mostly targeted EGFR signaling and the ERK MAPK signaling pathway, while drugs with sensitivity that was positively correlated with the CAPER score mostly targeted RTK signaling and cytoskeleton pathways.

## Discussion

In our study, using GWAS summary statistics of 15 cancers and the SuSiE package, we identified causal SNPs and 12 CAPER genes. SuSiE is a newly developed approach for genetic fine-mapping that quantifies the uncertainty of causal variables (Wang et al., 2020a). Compared with existing fine-mapping methods, such as CAVIAR (Hormozdiari et al., 2014), FINEMAP (Benner et al., 2016), and DAP-G (Wen et al., 2016; Lee et al., 2018), SuSiE has been demonstrated to have a fair degree of accuracy for computing PIPs and a much higher computation speed and higher power in distinguishing between causal variables and non-causal variables (Wang et al., 2020a). Then, we used the expression level of the CAPER genes in the TCGA training set to construct the CAPER score. We performed internal and external validation of the CAPER score using three validation sets, including the TCGA test set, IMvigor210, and GDSC. The results demonstrated the potential application of the CAPER score for therapeutic and prognostic purposes in cancer management.

Among the 54 causal SNPs obtained through fine-mapping, 29 were shown to have a significant eQTL effect on 8 potential causal genes across different tissues. A total of 324 eQTL pairs were extracted from GTEx and are listed in Supplementary Table S6. As expected, the association between all 12 CAPER genes and cancers have been demonstrated using a series of basic experimental and population-based studies. For example, *ATP6V1G2* is a ferroptosis-related gene that plays a primary role in metabolism and oxidative stress and is defined as a colorectal cancer prognosis biomarker (Huang et al., 2021; Shao et al., 2021). *DDX39B* is involved in the regulation of pre-mRNA splicing, nuclear export of mRNAs, and pre-ribosomal RNA translation, and may promote the genesis, development, and metastasis of multiple cancer types by regulating cell proliferation (Awasthi et al., 2018; Gu et al., 2020; Xu et al., 2020; He et al., 2021). *MYC* and *MYEOV* are two well-known oncogenes (Specht et al., 2004; Paglia et al., 2020). *MYC* can contribute to oncogenesis and immune evasion through various mechanisms, including the promotion of autonomous cell growth and proliferation, modulation of tumor–stroma interactions, and regulation of the host immune system (Dang, 2012; Paglia et al., 2020; Dhanasekaran et al., 2021; Lourenco et al., 2021), while the mechanism of action that underlies the function of *MYEOV* in cancer development and metastasis may enhance SOX9 transcriptional activity (Lawlor et al., 2010; Fang et al., 2019; Liang et al., 2020). Thus, both *MYC* and *MYEOV* have also been identified as potential immunotherapy targets (Fang et al., 2019; Duffy et al., 2021). *POU5F1B* can promote cancer oncogenesis by cooperating with *MYC* and is associated with poor prognosis in pancreatic ductal adenocarcinoma patients (Hayashi et al., 2015; Amantini et al., 2019). It may also promote HCC proliferation by activating AKT (Pan et al., 2018). *SLC6A19* has also been reported to be a potential biomarker that has a significantly low level of expression in patients with renal cancer (Zamora-Fuentes et al., 2020). In addition, a series of genetic variants in *MICA*, *MICB*, *NFKBIL1*, *SLC6A3*, *CLPTM1L,* and *TERT* have been found to be associated with the susceptibility and prognosis of different cancer types (Rafnar et al., 2009; Wang et al., 2009; Turnbull et al., 2010; Miki et al., 2011; Baek et al., 2018; Toledo-Stuardo et al., 2021). Meanwhile, using summary statistics of GETx data, we performed a colocalization analysis using the *coloc* package (v.5.1.0) (Giambartolomei et al., 2014). Using default settings, we found that the posterior probabilities of hypothesis 4 (PPH4: both the expression of *CLPTM1L* and MM are associated and share a single causal variant) are 0.986 and 0.985 for skin exposed to the Sun (lower leg) and skin not exposed to the Sun (suprapubic), respectively, while rs31490 had the largest PPH4 values of 0.541 and 0.624, respectively.

However, some well-known cancer-related genes, such as *TP53*, are not among the CAPER genes. For example, all108 SNPs of *TP53* were not significant in the 15 cancer summary statistics of the 15 types of cancer. There may be two probable reasons for this. First, the dysfunction of familiar genes may not have been caused by genetic variation. For example, the methylation of *TP53* has been regarded as a causal factor of leukemia (Saeed et al., 2019). Second, the limitation of sample size in the GWASs and its weak signal may result in SNPs from which common genes have been filtered out.

Furthermore, to verify the robustness and efficiency of the CAPER score, we applied it to TCGA test samples and the IMvigor210 cohort. As expected, patients in the high-risk group tended to have shorter survival and a worse TN stage, which together indicate a poor prognosis. The results also showed that samples in the high-risk group had a higher degree of immune infiltration and a lower differentiation ability, with a higher immune score and stemness score. The stemness score usually indicates the differentiation potential, and a loss of a differentiation ability and gain of stem-cell-like were reported to be the main signs of tumor progression (Seguin et al., 2015; Prasetyanti and Medema, 2017; Zhang et al., 2020); while immune infiltration was also reported to be correlated with the malignancy and prognosis of different types of cancer (Wang et al., 2020b; Wu et al., 2020; Zhang et al., 2020). In addition to the degree of infiltration, the composition of the infiltrating immune cell types in the high-risk and low-risk groups were also different, indicating a more complex difference in the tumor microenvironment between the two groups. Taken together, validation using the TCGA test samples and the IMvigor210 cohort indicated consistency of the CAPER score with clinical prognosis, proving its value in clinical translation.

Interestingly, some associations between genes and certain cancer types were not taken into account during the selection procedure but were detected during the validation procedure. For example, *MICB* is defined as the casual gene of CESC, PRAD, and UCEC (Table 2), but showed a high correlation with the tumor purity in four cancer types (KIRC, KIPP, LUAD, and MESO), which were not included in the selection step (Figures 5A,E). In addition, while THCA was not included in the selection step, its stemness showed a high level of correlation with the expression of *MICA*, *MYEOV*, *POU5F1B,* and *TRET* (Supplementary Figures S7A,C). This indicates the potential value of a pan-cancer analysis to identify novel associations.

In addition, we also found a link between the CAPER score and drug sensitivity using the GDSC database, which indicated the potentially extensible application of the CAPER score for the therapeutic and prognostic management of cancer. In particular, 81 drugs were found to be significantly correlated with the CAPER score, among which 67 showed a negative correlation with CAPER score, while only 16 showed higher sensitivity in the group with high CAPER score, indicating a limited selection of drugs available for the high-risk group.

In summary, we developed a CAPER score using a novel strategy based on fine-mapping. An extensive validation procedure was followed to confirm the robustness and efficiency of the CAPER score. Considering its potential usage in prognosis prediction and the identification of novel associations, we expect that this score may provide valuable information that can be used to better understand oncogenesis to guide management from a pan-cancer perspective.

## Data Availability

The original contributions presented in the study are included in the article/Supplementary Material. Further inquiries can be directed to the corresponding author.

## References

[B1] Albert F. W. Kruglyak L. (2015). The Role of Regulatory Variation in Complex Traits and Disease. Nat. Rev. Genet. 16, 197–212. 10.1038/nrg3891 25707927

[B2] Amantini C. Morelli M. B. Nabissi M. Piva F. Marinelli O. Maggi F. (2019). Expression Profiling of Circulating Tumor Cells in Pancreatic Ductal Adenocarcinoma Patients: Biomarkers Predicting Overall Survival. Front. Oncol. 9, 874. 10.3389/fonc.2019.00874 31552188 PMC6746928

[B3] Awasthi S. Chakrapani B. Mahesh A. Chavali P. L. Chavali S. Dhayalan A. (2018). DDX39B Promotes Translation through Regulation of Pre-ribosomal RNA Levels. RNA Biol. 15, 1157–1166. 10.1080/15476286.2018.1517011 30176153 PMC6284572

[B4] Aykul S. Martinez-Hackert E. (2016). Determination of Half-Maximal Inhibitory Concentration Using Biosensor-Based Protein Interaction Analysis. Anal. Biochem. 508, 97–103. 10.1016/j.ab.2016.06.025 27365221 PMC4955526

[B5] Baek I.-C. Shin D.-H. Choi E.-J. Kim H.-J. Yoon J.-H. Cho B.-S. (2018). Association of MICA and MICB Polymorphisms with the Susceptibility of Leukemia in Korean Patients. Blood Cancer J. 8, 58. 10.1038/s41408-018-0092-5 29895953 PMC5997647

[B6] Ballot E. Ladoire S. Routy B. Truntzer C. Ghiringhelli F. (2020). Tumor Infiltrating Lymphocytes Signature as a New Pan-Cancer Predictive Biomarker of Anti PD-1/pd-L1 Efficacy. Cancers 12, 2418. 10.3390/cancers12092418 PMC7564481 32858956

[B7] Bao Y. Wang L. Shi L. Yun F. Liu X. Chen Y. (2019). Transcriptome Profiling Revealed Multiple Genes and ECM-Receptor Interaction Pathways that May Be Associated with Breast Cancer. Cell Mol Biol Lett 24, 38. 10.1186/s11658-019-0162-0 31182966 PMC6554968

[B8] Barbeira A. N. Dickinson S. P. Dickinson S. P. Bonazzola R. Zheng J. Wheeler H. E. (2018). Exploring the Phenotypic Consequences of Tissue Specific Gene Expression Variation Inferred from GWAS Summary Statistics. Nat. Commun. 9, 1825. 10.1038/s41467-018-03621-1 29739930 PMC5940825

[B9] Benner C. Spencer C. C. A. Havulinna A. S. Salomaa V. Ripatti S. Pirinen M. (2016). FINEMAP: Efficient Variable Selection Using Summary Data from Genome-wide Association Studies. Bioinformatics 32, 1493–1501. 10.1093/bioinformatics/btw018 26773131 PMC4866522

[B10] Bray F. Ren J.-S. Masuyer E. Ferlay J. (2013). Global Estimates of Cancer Prevalence for 27 Sites in the Adult Population in 2008. Int. J. Cancer 132, 1133–1145. 10.1002/ijc.27711 22752881

[B11] Bulik-Sullivan B. K. Loh P.-R. Loh P.-R. Finucane H. K. Ripke S. Yang J. (2015). LD Score Regression Distinguishes Confounding from Polygenicity in Genome-wide Association Studies. Nat. Genet. 47, 291–295. 10.1038/ng.3211 25642630 PMC4495769

[B12] Chang K. Weinstein J. N. Collisson E. A. Mills G. B. Shaw K. R. Ozenberger B. A. (2013). The Cancer Genome Atlas Pan-Cancer Analysis Project. Nat. Genet. 45, 1113–1120. 10.1038/ng.2764 24071849 PMC3919969

[B13] Chen B. Khodadoust M. S. Liu C. L. Newman A. M. Alizadeh A. A. (2018). Profiling Tumor Infiltrating Immune Cells with CIBERSORT. Methods Mol. Biol. (Clifton, NJ) 1711, 243–259. 10.1007/978-1-4939-7493-1_12 PMC5895181 29344893

[B14] Ciriello G. Miller M. L. Aksoy B. A. Senbabaoglu Y. Schultz N. Sander C. (2013). Emerging Landscape of Oncogenic Signatures across Human Cancers. Nat. Genet. 45, 1127–1133. 10.1038/ng.2762 24071851 PMC4320046

[B15] Consortium G. P. Auton A. Brooks L. D. Durbin R. M. Garrison E. P. Kang H. M. (2015). A Global Reference for Human Genetic Variation. Nature 526, 68–74. 10.1038/nature15393 26432245 PMC4750478

[B16] Craven K. E. Gökmen-Polar Y. Badve S. S. (2021). CIBERSORT Analysis of TCGA and METABRIC Identifies Subgroups with Better Outcomes in Triple Negative Breast Cancer. Sci. Rep. 11 (1), 1–19. 10.1038/s41598-021-83913-7 33633150 PMC7907367

[B17] Dang C. V. (2012). MYC on the Path to Cancer. Cell 149, 22–35. 10.1016/j.cell.2012.03.003 22464321 PMC3345192

[B18] De Los Campos G. Vazquez A. I. Hsu S. Lello L. (2018). Complex-Trait Prediction in the Era of Big Data. Trends Genet. 34, 746–754. 10.1016/j.tig.2018.07.004 30139641 PMC6150788

[B19] Demircioğlu D. Cukuroglu E. Kindermans M. Nandi T. Calabrese C. Fonseca N. A. (2019). A Pan-Cancer Transcriptome Analysis Reveals Pervasive Regulation through Alternative Promoters. Cell 178, 1465–e17. e1417. 10.1016/j.cell.2019.08.018 31491388

[B20] Deng Y. Huang Z. Xu Y. Jin J. Zhuo W. Zhang C. (2014). MiR-215 Modulates Gastric Cancer Cell Proliferation by Targeting RB1. Cancer Lett. 342, 27–35. 10.1016/j.canlet.2013.08.033 23981575

[B21] Dhanasekaran R. Deutzmann A. Mahauad-Fernandez W. D. Hansen A. S. Gouw A. M. Felsher D. W. (2021). The MYC Oncogene-The Grand Orchestrator of Cancer Growth and Immune Evasion. Nat. Rev. Clin. Oncol. 2021, 1–14. 10.1038/s41571-021-00549-2 PMC9083341 34508258

[B22] Duffy M. J. O'grady S. Tang M. Crown J. (2021). MYC as a Target for Cancer Treatment. Cancer Treat. Rev. 94, 102154. 10.1016/j.ctrv.2021.102154 33524794

[B23] Durinck S. Spellman P. T. Birney E. Huber W. (2009). Mapping Identifiers for the Integration of Genomic Datasets with the R/Bioconductor Package biomaRt. Nat. Protoc. 4, 1184–1191. 10.1038/nprot.2009.97 19617889 PMC3159387

[B24] Fadista J. Manning A. K. Florez J. C. Groop L. (2016). The (In)famous GWAS P-Value Threshold Revisited and Updated for Low-Frequency Variants. Eur. J. Hum. Genet. 24, 1202–1205. 10.1038/ejhg.2015.269 26733288 PMC4970684

[B25] Fang L. Wu S. Zhu X. Cai J. Wu J. He Z. (2019). MYEOV Functions as an Amplified Competing Endogenous RNA in Promoting Metastasis by Activating TGF-β Pathway in NSCLC. Oncogene 38, 896–912. 10.1038/s41388-018-0484-9 30181549 PMC6756124

[B26] Frost F. G. Cherukuri P. F. Milanovich S. Boerkoel C. F. (2020). Pan‐cancer RNA‐seq Data Stratifies Tumours by Some Hallmarks of Cancer. J. Cel Mol Med 24, 418–430. 10.1111/jcmm.14746 PMC6933344 31730267

[B27] Gallagher M. D. Chen-Plotkin A. S. (2018). The Post-GWAS Era: From Association to Function. Am. J. Hum. Genet. 102, 717–730. 10.1016/j.ajhg.2018.04.002 29727686 PMC5986732

[B28] Giambartolomei C. Vukcevic D. Schadt E. E. Franke L. Hingorani A. D. Wallace C. (2014). Bayesian Test for Colocalisation between Pairs of Genetic Association Studies Using Summary Statistics. Plos Genet. 10, e1004383. 10.1371/journal.pgen.1004383 24830394 PMC4022491

[B29] Goldman M. Craft B. Brooks A. Zhu J. Haussler D. (2018). The UCSC Xena Platform for Cancer Genomics Data Visualization and Interpretation. biorxiv 2018, 326470. 10.1101/326470

[B30] Gu H.-Y. Zhang C. Guo J. Yang M. Zhong H.-C. Jin W. (2020). Risk Score Based on Expression of Five Novel Genes Predicts Survival in Soft Tissue Sarcoma. Aging 12, 3807–3827. 10.18632/aging.102847 32084007 PMC7066896

[B31] Gusev A. Ko A. Shi H. Bhatia G. Chung W. Penninx B. W. J. H. (2016). Integrative Approaches for Large-Scale Transcriptome-wide Association Studies. Nat. Genet. 48, 245–252. 10.1038/ng.3506 26854917 PMC4767558

[B32] Hayashi H. Arao T. Togashi Y. Kato H. Fujita Y. De Velasco M. A. (2015). The OCT4 Pseudogene POU5F1B Is Amplified and Promotes an Aggressive Phenotype in Gastric Cancer. Oncogene 34, 199–208. 10.1038/onc.2013.547 24362523

[B33] He C. Li A. Lai Q. Ding J. Yan Q. Liu S. (2021). The DDX39B/FUT3/TGFβR-I axis Promotes Tumor Metastasis and EMT in Colorectal Cancer. Cell Death Dis 12, 74. 10.1038/s41419-020-03360-6 33436563 PMC7803960

[B34] Hormozdiari F. Kostem E. Kang E. Y. Pasaniuc B. Eskin E. (2014). Identifying Causal Variants at Loci with Multiple Signals of Association. Genetics 198, 497–508. 10.1534/genetics.114.167908 25104515 PMC4196608

[B35] Huang C. Zhao J. Zhu Z. (2021). Prognostic Nomogram of Prognosis-Related Genes and Clinicopathological Characteristics to Predict the 5-Year Survival Rate of Colon Cancer Patients. Front. Surg. 8. 10.3389/fsurg.2021.681721 PMC8242155 34222322

[B36] Kandoth C. Mclellan M. D. Vandin F. Ye K. Niu B. Lu C. (2013). Mutational Landscape and Significance across 12 Major Cancer Types. Nature 502, 333–339. 10.1038/nature12634 24132290 PMC3927368

[B37] Kim S.-K. Kim H.-J. Park J.-L. Heo H. Kim S.-Y. Lee S.-I. (2020). Identification of a Molecular Signature of Prognostic Subtypes in Diffuse-type Gastric Cancer. Gastric Cancer 23, 473–482. 10.1007/s10120-019-01029-4 31773340 PMC7165151

[B38] Lappalainen T. Sammeth M. Sammeth M. Friedländer M. R. ‘t Hoen P. A. C. Monlong J. (2013). Transcriptome and Genome Sequencing Uncovers Functional Variation in Humans. Nature 501, 506–511. 10.1038/nature12531 24037378 PMC3918453

[B39] Lawlor G. Doran P. P. Macmathuna P. Murray D. W. (2010). MYEOV (Myeloma Overexpressed Gene) Drives colon Cancer Cell Migration and Is Regulated by PGE2. J. Exp. Clin. Cancer Res. 29, 81–85. 10.1186/1756-9966-29-81 20569498 PMC2904283

[B40] Lee Y. Luca F. Pique-Regi R. Wen X. (2018). Bayesian Multi-SNP Genetic Association Analysis: Control of FDR and Use of Summary Statistics. bioRxiv 2018, 316471. 10.1371/journal.pgen.1007856

[B41] Leo P. J. Madeleine M. M. Wang S. Schwartz S. M. Newell F. Pettersson-Kymmer U. (2017). Defining the Genetic Susceptibility to Cervical Neoplasia-A Genome-wide Association Study. Plos Genet. 13, e1006866. 10.1371/journal.pgen.1006866 28806749 PMC5570502

[B42] Li D. Duell E. J. Yu K. Risch H. A. Olson S. H. Kooperberg C. (2012). Pathway Analysis of Genome-wide Association Study Data Highlights Pancreatic Development Genes as Susceptibility Factors for Pancreatic Cancer. Carcinogenesis 33, 1384–1390. 10.1093/carcin/bgs151 22523087 PMC3405651

[B43] Li R. Yin Y.-H. Ji X.-L. Liu X. Li J.-P. Qu Y.-Q. (2021). Pan-Cancer Prognostic, Immunity, Stemness, and Anticancer Drug Sensitivity Characterization of N6-Methyladenosine RNA Modification Regulators in Human Cancers. Front. Mol. Biosciences 8, 644620. 10.3389/fmolb.2021.644620 PMC8211991 34150845

[B44] Liang E. Lu Y. Shi Y. Zhou Q. Zhi F. (2020). MYEOV Increases HES1 Expression and Promotes Pancreatic Cancer Progression by Enhancing SOX9 Transactivity. Oncogene 39, 6437–6450. 10.1038/s41388-020-01443-4 32879444

[B45] Liberzon A. Birger C. Thorvaldsdóttir H. Ghandi M. Mesirov J. P. Tamayo P. (2015). The Molecular Signatures Database Hallmark Gene Set Collection. Cel Syst. 1, 417–425. 10.1016/j.cels.2015.12.004 PMC4707969 26771021

[B46] Liberzon A. Subramanian A. Pinchback R. Thorvaldsdóttir H. Tamayo P. Mesirov J. P. (2011). Molecular Signatures Database (MSigDB) 3.0. Bioinformatics 27, 1739–1740. 10.1093/bioinformatics/btr260 21546393 PMC3106198

[B47] Liu J. Mccleland M. Stawiski E. W. Gnad F. Mayba O. Haverty P. M. (2014). Integrated Exome and Transcriptome Sequencing Reveals ZAK Isoform Usage in Gastric Cancer. Nat. Commun. 5, 3830. 10.1038/ncomms4830 24807215 PMC4024760

[B48] Liu J. N. Kong X. S. Huang T. Wang R. Li W. Chen Q. F. (2020). Clinical Implications of Aberrant PD-1 and CTLA4 Expression for Cancer Immunity and Prognosis: A Pan-Cancer Study. Front. Immunol. 11, 2048. 10.3389/fimmu.2020.02048 33072070 PMC7539667

[B49] Lourenco C. Resetca D. Redel C. Lin P. Macdonald A. S. Ciaccio R. (2021). MYC Protein Interactors in Gene Transcription and Cancer. Nat. Rev. Cancer 21, 579–591. 10.1038/s41568-021-00367-9 34188192

[B50] Malta T. M. Sokolov A. Gentles A. J. Burzykowski T. Poisson L. Weinstein J. N. (2018). Machine Learning Identifies Stemness Features Associated with Oncogenic Dedifferentiation. Cell 173, 338–e15. e315. 10.1016/j.cell.2018.03.034 29625051 PMC5902191

[B51] Mancuso N. Freund M. K. Johnson R. Shi H. Kichaev G. Gusev A. (2019). Probabilistic fine-mapping of Transcriptome-wide Association Studies. Nat. Genet. 51, 675–682. 10.1038/s41588-019-0367-1 30926970 PMC6619422

[B52] Mariathasan S. Turley S. J. Nickles D. Castiglioni A. Yuen K. Wang Y. (2018). TGFβ Attenuates Tumour Response to PD-L1 Blockade by Contributing to Exclusion of T Cells. Nature 554, 544–548. 10.1038/nature25501 29443960 PMC6028240

[B53] Maurano M. T. Humbert R. Rynes E. Thurman R. E. Haugen E. Wang H. (2012). Systematic Localization of Common Disease-Associated Variation in Regulatory DNA. Science 337, 1190–1195. 10.1126/science.1222794 22955828 PMC3771521

[B54] Miki D. Ochi H. Hayes C. N. Abe H. Yoshima T. Aikata H. (2011). Variation in the DEPDC5 Locus Is Associated with Progression to Hepatocellular Carcinoma in Chronic Hepatitis C Virus Carriers. Nat. Genet. 43, 797–800. 10.1038/ng.876 21725309

[B55] Nakagawa H. Fujita M. (2018). Whole Genome Sequencing Analysis for Cancer Genomics and Precision Medicine. Cancer Sci. 109, 513–522. 10.1111/cas.13505 29345757 PMC5834793

[B56] Nawy T. (2018). A Pan-Cancer Atlas. Nat. Methods 15, 407. 10.1038/s41592-018-0020-4 29855579

[B57] O'Mara T. A. Glubb D. M. Amant F. Annibali D. Ashton K. Attia J. (2018). Identification of Nine New Susceptibility Loci for Endometrial Cancer. Nat. Commun. 9 (1), 1–12. 10.1038/s41467-018-05427-7 30093612 PMC6085317

[B58] Paglia S. Sollazzo M. Di Giacomo S. Strocchi S. Grifoni D. (2020). Exploring MYC Relevance to Cancer Biology from the Perspective of Cell Competition. Semin. Cancer Biol. 63, 49–59. 10.1016/j.semcancer.2019.05.009 31102666

[B59] Pan Y. Zhan L. Chen L. Zhang H. Sun C. Xing C. (2018). POU5F1B Promotes Hepatocellular Carcinoma Proliferation by Activating AKT. Biomed. Pharmacother. 100, 374–380. 10.1016/j.biopha.2018.02.023 29454285

[B60] Pei J. Wang Y. Li Y. (2020). Identification of Key Genes Controlling Breast Cancer Stem Cell Characteristics via Stemness Indices Analysis. J. Transl Med. 18, 74–15. 10.1186/s12967-020-02260-9 32050983 PMC7014665

[B61] Peng J. Guan J. Hui W. Shang X. (2021). A Novel Subnetwork Representation Learning Method for Uncovering Disease-Disease Relationships. Methods 192, 77–84. 10.1016/j.ymeth.2020.09.002 32946974

[B62] Peng J. Hui W. Li Q. Chen B. Hao J. Jiang Q. (2019). A Learning-Based Framework for miRNA-Disease Association Identification Using Neural Networks. Bioinformatics 35, 4364–4371. 10.1093/bioinformatics/btz254 30977780

[B63] Phelan C. M. Kuchenbaecker K. B. Tyrer J. P. Kar S. P. Lawrenson K. Winham S. J. (2017). Identification of 12 New Susceptibility Loci for Different Histotypes of Epithelial Ovarian Cancer. Nat. Genet. 49, 680–691. 10.1038/ng.3826 28346442 PMC5612337

[B64] Prasetyanti P. R. Medema J. P. (2017). Intra-tumor Heterogeneity from a Cancer Stem Cell Perspective. Mol. Cancer 16, 41. 10.1186/s12943-017-0600-4 28209166 PMC5314464

[B65] Priestley P. Baber J. Lolkema M. P. Steeghs N. De Bruijn E. Shale C. (2019). Pan-cancer Whole-Genome Analyses of Metastatic Solid Tumours. Nature 575, 210–216. 10.1038/s41586-019-1689-y 31645765 PMC6872491

[B66] Rafnar T. Sulem P. Stacey S. N. Geller F. Gudmundsson J. Sigurdsson A. (2009). Sequence Variants at the TERT-Clptm1l Locus Associate with many Cancer Types. Nat. Genet. 41, 221–227. 10.1038/ng.296 19151717 PMC4525478

[B67] Rashkin S. R. Graff R. E. Kachuri L. Thai K. K. Alexeeff S. E. Blatchins M. A. (2020). Pan-cancer Study Detects Genetic Risk Variants and Shared Genetic Basis in Two Large Cohorts. Nat. Commun. 11 (1), 1–14. 10.1038/s41467-020-18246-6 32887889 PMC7473862

[B68] Ritchie M. E. Phipson B. Wu D. Hu Y. Law C. W. Shi W. (2015). Limma powers Differential Expression Analyses for RNA-Sequencing and Microarray Studies. Nucleic Acids Res. 43, e47. 10.1093/nar/gkv007 25605792 PMC4402510

[B69] Rodriguez-Martin B. Alvarez E. G. Baez-Ortega A. Zamora J. Supek F. Demeulemeester J. (2020). Pan-cancer Analysis of Whole Genomes Identifies Driver Rearrangements Promoted by LINE-1 Retrotransposition. Nat. Genet. 52, 306–319. 10.1038/s41588-019-0562-0 32024998 PMC7058536

[B70] Saeed W. H. Eissa A. A. Al-Doski A. A. (2019). Impact of TP53 Gene Promoter Methylation on Chronic Lymphocytic Leukemia Pathogenesis and Progression. Jbm Vol. 10, 399–404. 10.2147/jbm.s221707 PMC6883927 31819692

[B71] Schaid D. J. Chen W. Larson N. B. (2018). From Genome-wide Associations to Candidate Causal Variants by Statistical fine-mapping. Nat. Rev. Genet. 19, 491–504. 10.1038/s41576-018-0016-z 29844615 PMC6050137

[B72] Schumacher F. R. Al Olama A. A. Berndt S. I. Benlloch S. Ahmed M. Saunders E. J. (2018). Association Analyses of More Than 140,000 Men Identify 63 New Prostate Cancer Susceptibility Loci. Nat. Genet. 50, 928–936. 10.1038/s41588-018-0142-8 29892016 PMC6568012

[B73] Seguin L. Desgrosellier J. S. Weis S. M. Cheresh D. A. (2015). Integrins and Cancer: Regulators of Cancer Stemness, Metastasis, and Drug Resistance. Trends Cel Biol. 25, 234–240. 10.1016/j.tcb.2014.12.006 PMC4380531 25572304

[B74] Shao Y. Jia H. Huang L. Li S. Wang C. Aikemu B. (2021). An Original Ferroptosis-Related Gene Signature Effectively Predicts the Prognosis and Clinical Status for Colorectal Cancer Patients. Front. Oncol. 11, 711776. 10.3389/fonc.2021.711776 34249766 PMC8264263

[B75] Shukla S. Evans J. R. Malik R. Feng F. Y. Dhanasekaran S. M. Cao X. (2016). Development of a RNA-Seq Based Prognostic Signature in Lung Adenocarcinoma. J. Natl. Cancer Inst. Natl. Cancer Inst. 109, djw200. 10.1093/jnci/djw200 PMC5051943 27707839

[B76] Specht K. Haralambieva E. Bink K. Kremer M. Mandl-Weber S. Koch I. (2004). Different Mechanisms of Cyclin D1 Overexpression in Multiple Myeloma Revealed by Fluorescence *In Situ* Hybridization and Quantitative Analysis of mRNA Levels. Blood 104, 1120–1126. 10.1182/blood-2003-11-3837 15090460

[B77] Steen C. B. Liu C. L. Alizadeh A. A. Newman A. M. (2020). “Profiling Cell Type Abundance and Expression in Bulk Tissues with CIBERSORTx,” in Stem Cell Transcriptional Networks. Editor Kidder B. L. (New York, United States: Springer), 135–157. 10.1007/978-1-0716-0301-7_7 PMC7695353 31960376

[B78] Subramanian A. Tamayo P. Mootha V. K. Mukherjee S. Ebert B. L. Gillette M. A. (2005). Gene Set Enrichment Analysis: A Knowledge-Based Approach for Interpreting Genome-wide Expression Profiles. Proc. Natl. Acad. Sci. 102, 15545–15550. 10.1073/pnas.0506580102 16199517 PMC1239896

[B79] Tam V. Patel N. Turcotte M. Bossé Y. Paré G. Meyre D. (2019). Benefits and Limitations of Genome-wide Association Studies. Nat. Rev. Genet. 20, 467–484. 10.1038/s41576-019-0127-1 31068683

[B80] Tarver T. (2012). Cancer Facts & Figures 2012. American Cancer Society (ACS). J. Consumer Health Internet 16, 366–367. 10.1080/15398285.2012.701177

[B81] Thorsson V. Gibbs D. L. Brown S. D. Wolf D. Bortone D. S. Ou Yang T. H. (2018). The Immune Landscape of Cancer. Immunity 48, 812–e14. e814. 10.1016/j.immuni.2018.03.023 29628290 PMC5982584

[B82] Toledo-Stuardo K. Ribeiro C. H. Canals A. Morales M. Gárate V. Rodríguez-Siza J. (2021). Major Histocompatibility Complex Class I-Related Chain A (MICA) Allelic Variants Associate with Susceptibility and Prognosis of Gastric Cancer. Front. Immunol. 12, 645528. 10.3389/fimmu.2021.645528 33868281 PMC8045969

[B83] Tomczak K. Czerwińska P. Wiznerowicz M. (2015). The Cancer Genome Atlas (TCGA): an Immeasurable Source of Knowledge. Contemp. Oncol. (Pozn) 19, A68–A77. 10.5114/wo.2014.47136 25691825 PMC4322527

[B84] Turnbull C. Rapley E. A. Rapley E. A. Seal S. Pernet D. Renwick A. (2010). Variants Near DMRT1, TERT and ATF7IP Are Associated with Testicular Germ Cell Cancer. Nat. Genet. 42, 604–607. 10.1038/ng.607 20543847 PMC3773909

[B85] Vargas A. J. Harris C. C. (2016). Biomarker Development in the Precision Medicine Era: Lung Cancer as a Case Study. Nat. Rev. Cancer 16, 525–537. 10.1038/nrc.2016.56 27388699 PMC6662593

[B86] Visscher P. M. Wray N. R. Zhang Q. Sklar P. Mccarthy M. I. Brown M. A. (2017). 10 Years of GWAS Discovery: Biology, Function, and Translation. Am. J. Hum. Genet. 101, 5–22. 10.1016/j.ajhg.2017.06.005 28686856 PMC5501872

[B87] Wainberg M. Sinnott-Armstrong N. Mancuso N. Barbeira A. N. Knowles D. A. Golan D. (2019). Opportunities and Challenges for Transcriptome-wide Association Studies. Nat. Genet. 51, 592–599. 10.1038/s41588-019-0385-z 30926968 PMC6777347

[B88] Wang G. Sarkar A. Carbonetto P. Stephens M. (2020a). A Simple New Approach to Variable Selection in Regression, with Application to Genetic fine Mapping. J. R. Stat. Soc. B 82, 1273–1300. 10.1111/rssb.12388 PMC10201948 37220626

[B89] Wang S. Xiong Y. Zhang Q. Su D. Yu C. Cao Y. (2020b). Clinical Significance and Immunogenomic Landscape Analyses of the Immune Cell Signature Based Prognostic Model for Patients with Breast Cancer. Brief Bioinform 22. 10.1093/bib/bbaa311 33302293

[B90] Wang S. S. Purdue M. P. Cerhan J. R. Zheng T. Menashe I. Armstrong B. K. (2009). Common Gene Variants in the Tumor Necrosis Factor (TNF) and TNF Receptor Superfamilies and NF-kB Transcription Factors and Non-hodgkin Lymphoma Risk. PLOS ONE 4, e5360. 10.1371/journal.pone.0005360 19390683 PMC2669130

[B91] Wen X. Lee Y. Luca F. Pique-Regi R. (2016). Efficient Integrative Multi-SNP Association Analysis via Deterministic Approximation of Posteriors. Am. J. Hum. Genet. 98, 1114–1129. 10.1016/j.ajhg.2016.03.029 27236919 PMC4908152

[B92] Westra H.-J. Peters M. J. Esko T. Yaghootkar H. Schurmann C. Kettunen J. (2013). Systematic Identification of Trans eQTLs as Putative Drivers of Known Disease Associations. Nat. Genet. 45, 1238–1243. 10.1038/ng.2756 24013639 PMC3991562

[B93] Wu C. Pan W. (2020). A Powerful fine-mapping Method for Transcriptome-wide Association Studies. Hum. Genet. 139, 199–213. 10.1007/s00439-019-02098-2 31844974 PMC6983348

[B94] Wu F. Wang Z. L. Wang K. Y. Li G. Z. Chai R. C. Liu Y. Q. (2020). Classification of Diffuse Lower‐grade Glioma Based on Immunological Profiling. Mol. Oncol. 14, 2081–2095. 10.1002/1878-0261.12707 32392361 PMC7463381

[B95] Xu Z. Li X. Li H. Nie C. Liu W. Li S. (2020). Suppression of DDX39B Sensitizes Ovarian Cancer Cells to DNA-Damaging Chemotherapeutic Agents via Destabilizing BRCA1 mRNA. Oncogene 39, 7051–7062. 10.1038/s41388-020-01482-x 32989256

[B96] Xu Z. Wu C. Wei P. Pan W. (2017). A Powerful Framework for Integrating eQTL and GWAS Summary Data. Genetics 207, 893–902. 10.1534/genetics.117.300270 28893853 PMC5676241

[B97] Yang W. Soares J. Greninger P. Edelman E. J. Lightfoot H. Forbes S. (2012). Genomics of Drug Sensitivity in Cancer (GDSC): a Resource for Therapeutic Biomarker Discovery in Cancer Cells. Nucleic Acids Res. 41, D955–D961. 10.1093/nar/gks1111 23180760 PMC3531057

[B98] Yoshihara K. Shahmoradgoli M. Martínez E. Vegesna R. Kim H. Torres-Garcia W. (2013). Inferring Tumour Purity and Stromal and Immune Cell Admixture from Expression Data. Nat. Commun. 4 (1), 1–11. 10.1038/ncomms3612 PMC3826632 24113773

[B99] Zamora-Fuentes J. M. Hernández-Lemus E. Espinal-Enríquez J. (2020). Gene Expression and Co-expression Networks Are Strongly Altered through Stages in Clear Cell Renal Carcinoma. Front. Genet. 11, 578679. 10.3389/fgene.2020.578679 33240325 PMC7669746

[B100] Zeng P. Dai J. Jin S. Zhou X. (2021). Aggregating Multiple Expression Prediction Models Improves the Power of Transcriptome-wide Association Studies. Hum. Mol. Genet. 30, 939–951. 10.1093/hmg/ddab056 33615361

[B101] Zhang C. Chen T. Li Z. Liu A. Xu Y. Gao Y. (2020). Depiction of Tumor Stemlike Features and Underlying Relationships with hazard Immune Infiltrations Based on Large Prostate Cancer Cohorts. Brief Bioinform 22. 10.1093/bib/bbaa211 32856039

[B102] Zhang W. Bouchard G. Yu A. Shafiq M. Jamali M. Shrager J. B. (2018). GFPT2-Expressing Cancer-Associated Fibroblasts Mediate Metabolic Reprogramming in Human Lung Adenocarcinoma. Cancer Res. 78, 3445–3457. 10.1158/0008-5472.CAN-17-2928 29760045 PMC6030462

[B103] Zhou W. Nielsen J. B. Fritsche L. G. Dey R. Gabrielsen M. E. Wolford B. N. (2018). Efficiently Controlling for Case-Control Imbalance and Sample Relatedness in Large-Scale Genetic Association Studies. Nat. Genet. 50, 1335–1341. 10.1038/s41588-018-0184-y 30104761 PMC6119127

