# Supplementary material for: Construction and Clinical Translation of Causal Pan-Cancer Gene Score Across Cancer Types
Source: Front Genet. 2021 Dec 23;12:784775. doi: 10.3389/fgene.2021.784775 (PMC8733729; doi:10.3389/fgene.2021.784775)
Supplement: Supplementary file 1 [file DataSheet1.docx]

Supplementary Material

## Supplementary Figures


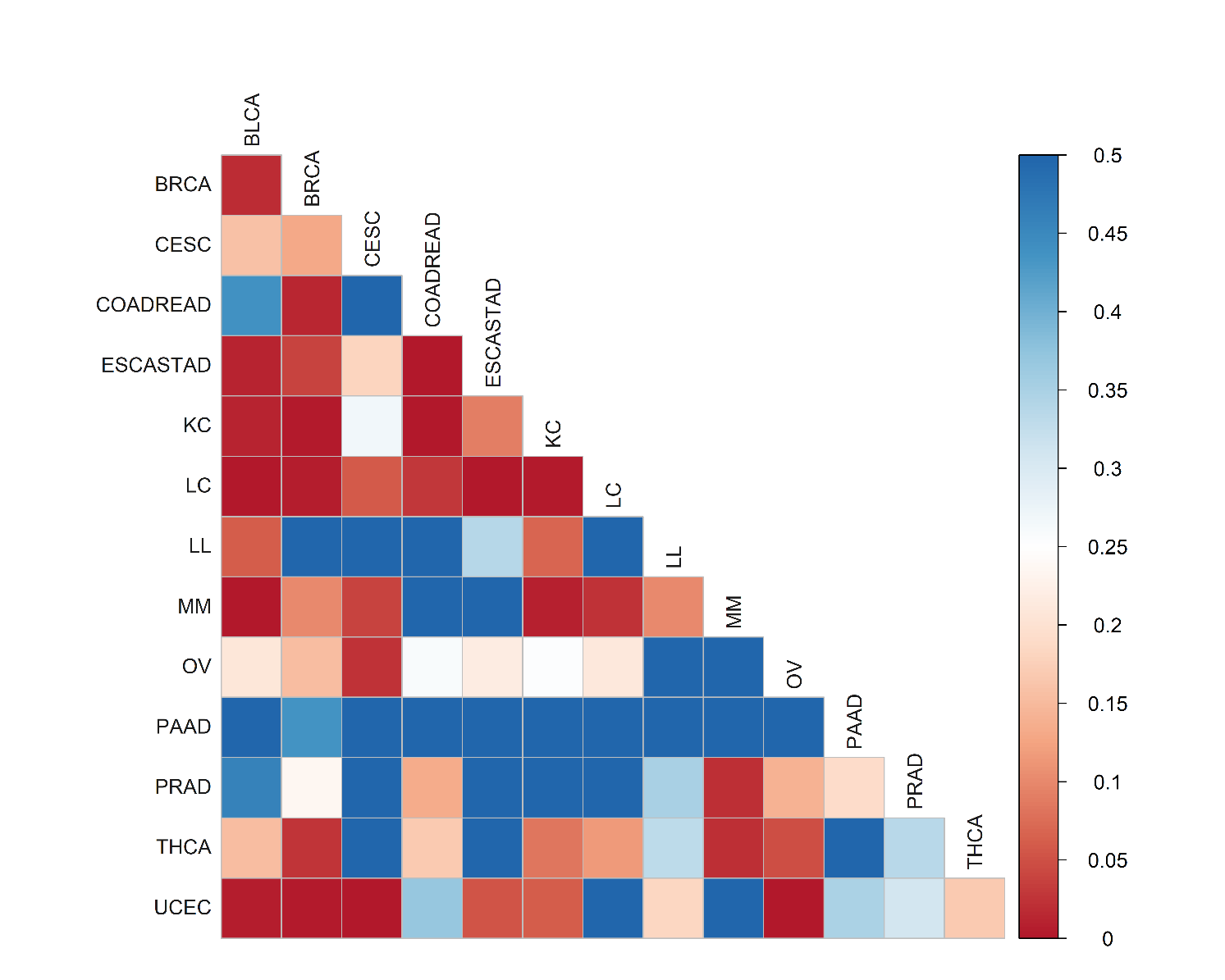


**Supplementary Figure S1.** **The genetic correlation among 14 cancer types using LDSC**. The correlation matrix plot shows the *P* value of genetic correlation coefficient. Note that results on OCPC are not shown for LDSC unable to estimate genetic correlations, likely due to small sample sizes and low heritability.

**Supplementary Figure S2.** **ROC of the multivariate Cox regression conducted on TCGA training set.** We estimated AUCs for one (green), three (red), and five (blue) years.


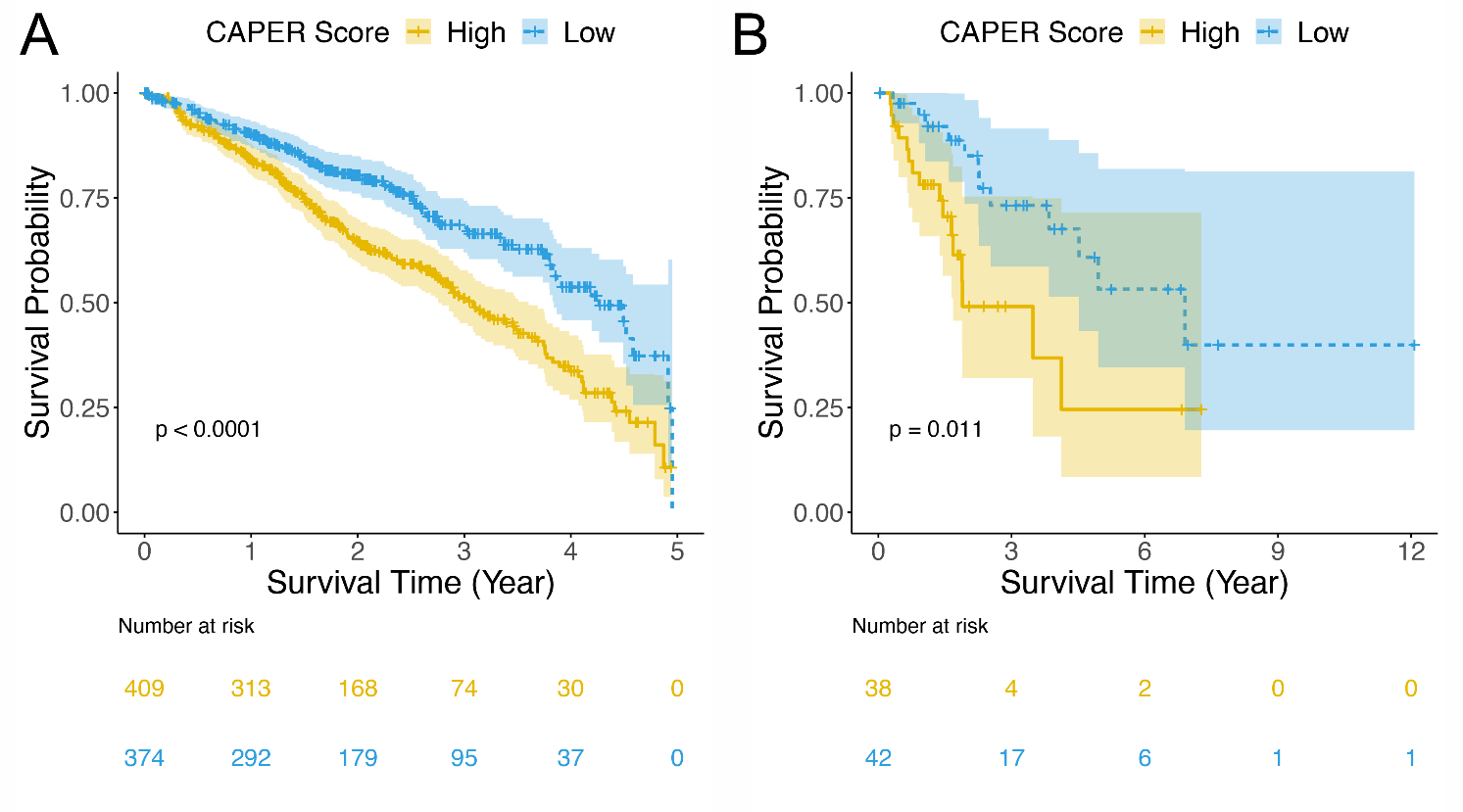


**Supplementary Figure S3. K-M curve of the high- and low-CAPER score groups. (A)** Survival time≤5 years **(B)** Random 80 samples


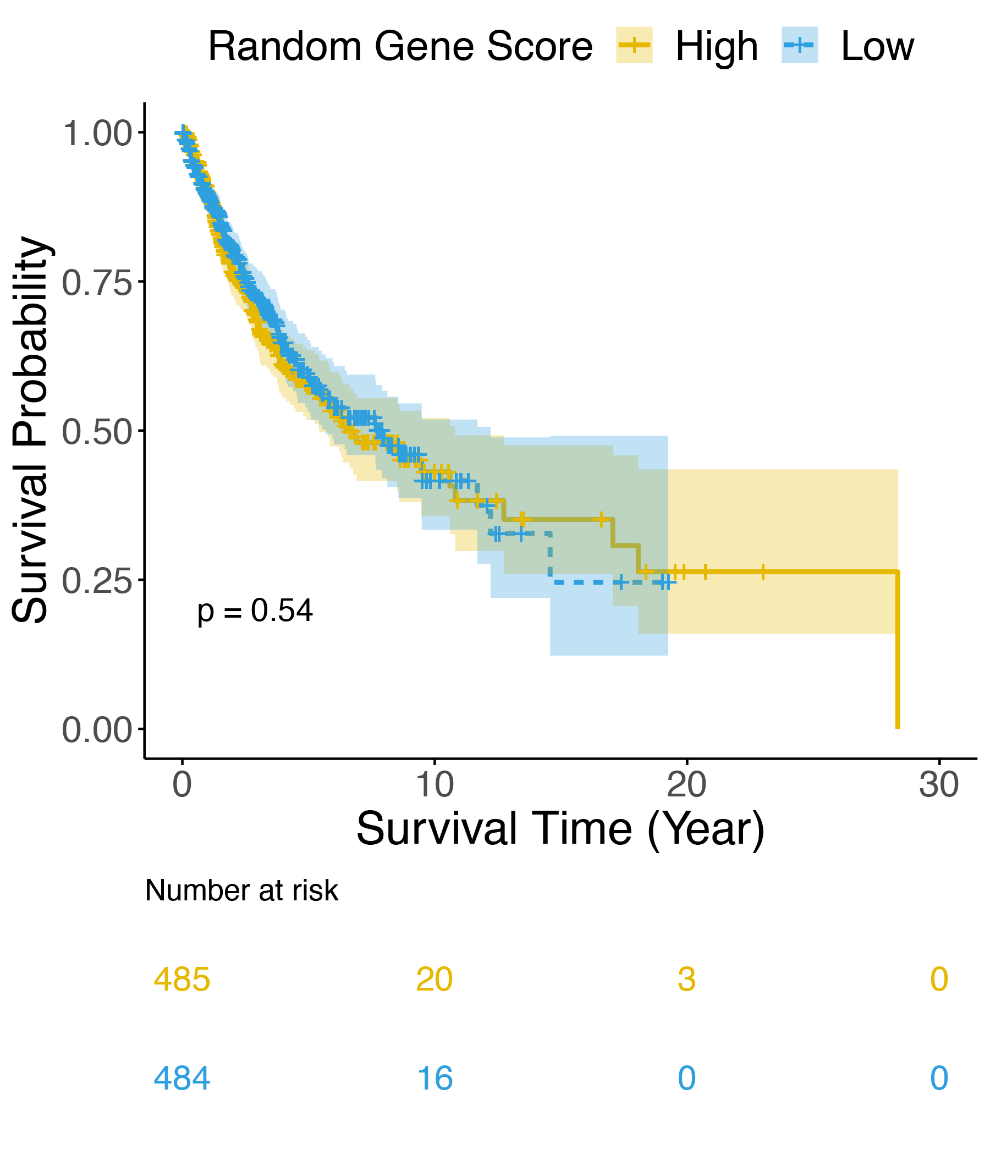


**Supplementary Figure S4. K-M curve of the high- and low- random gene score groups.**


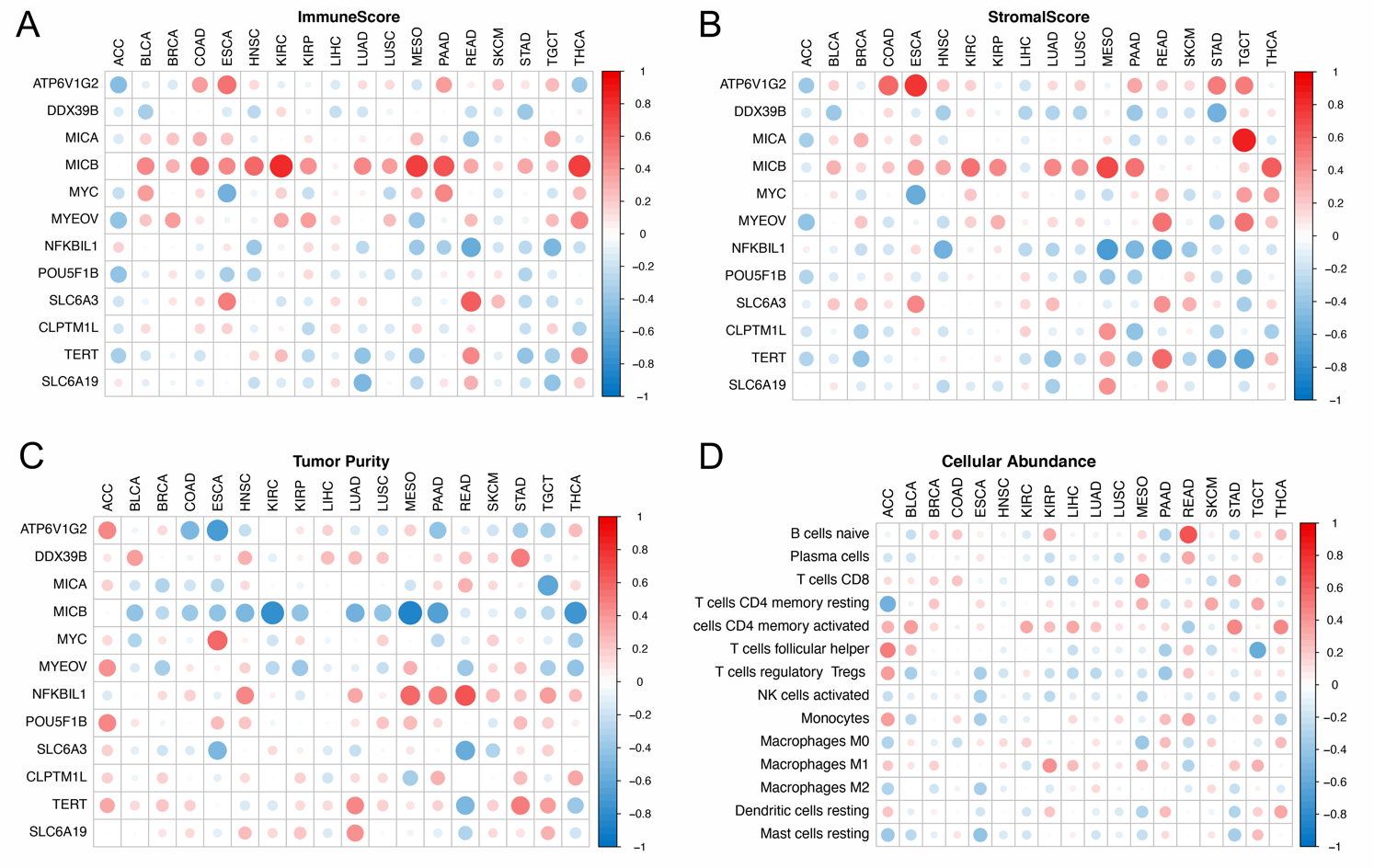


**Supplementary Figure S5. Summary of the correlation between TME variables and CAPER gene expression using TCGA test set. (A)** The results of the Spearman correlation between single gene expression and immune score in 21 types of cancer. **(B)** The results of the Spearman correlation between single gene expression and stromal score in 18 types of cancer. **(C)** The results of the Spearman correlation between single gene expression and tumor purity in 18 types of cancer. **(D)** The Spearman correlation of CAPER score and cellular abundance in 18 types of cancer.


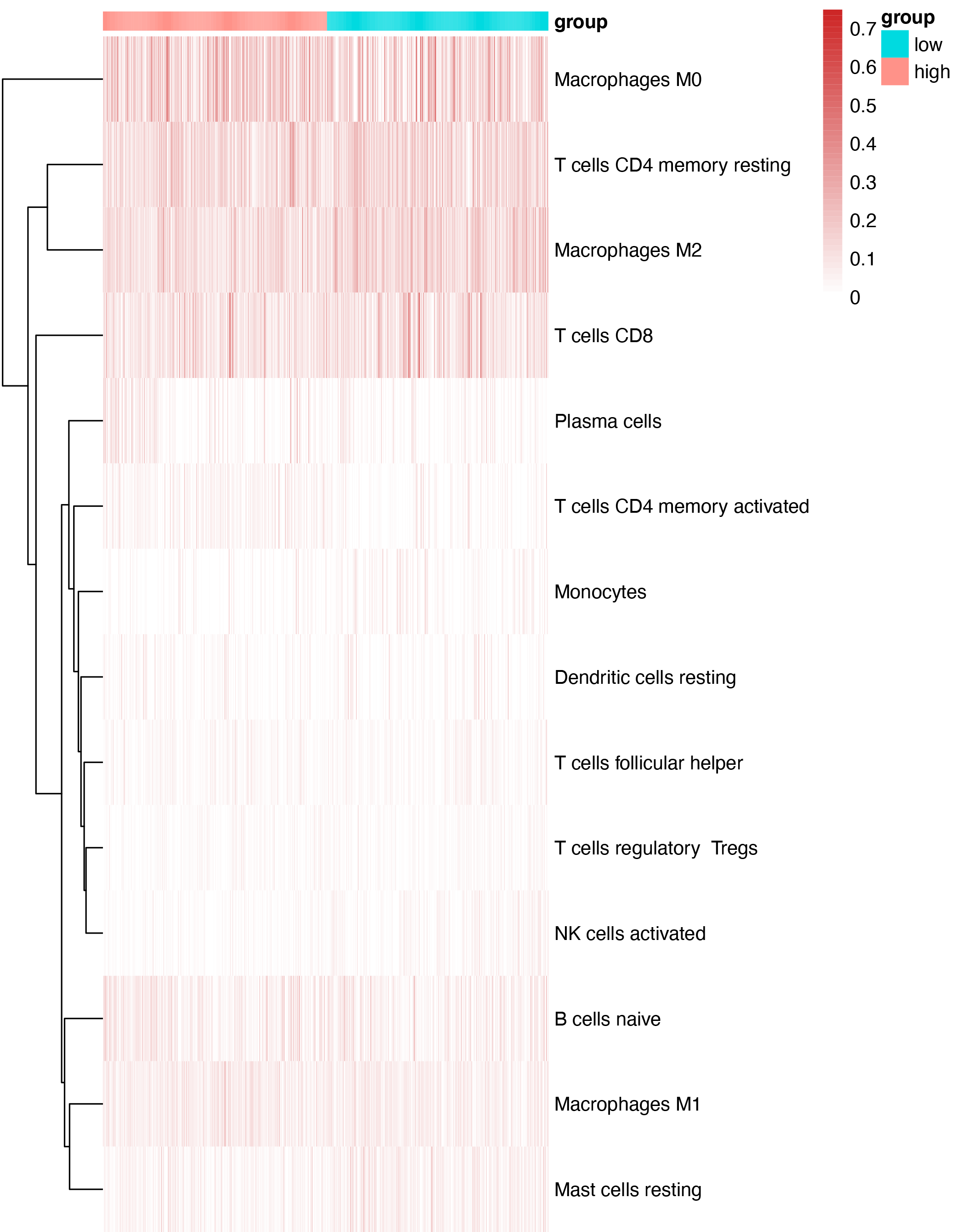


**Supplementary Figure S6.** **The heatmap of immune cell abundance**


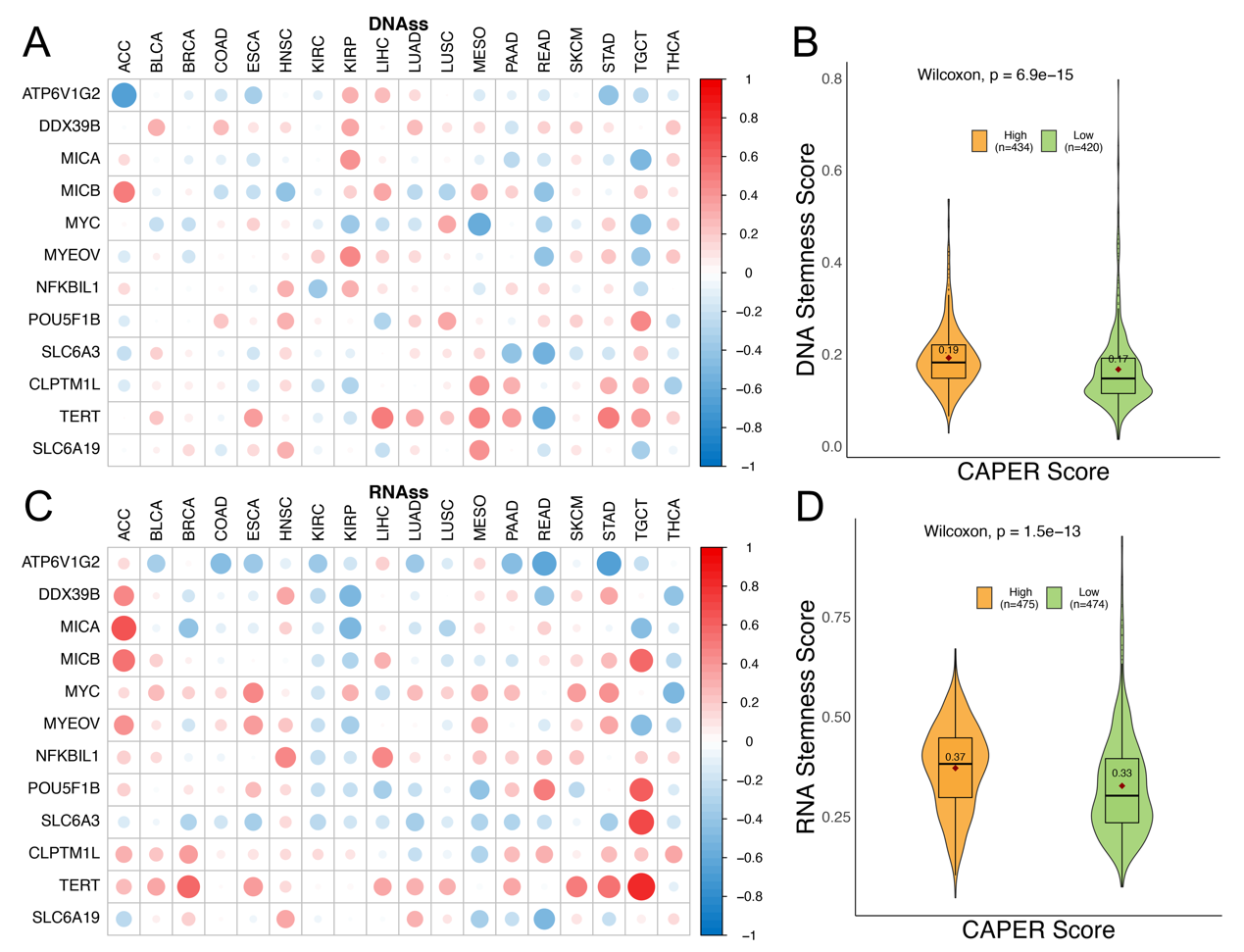


**Supplementary Figure S7. Summary of the correlation between stemness score and CAPER score or CAPER gene expression using TCGA test set. (A)** The results of the Spearman correlation between single gene expression and DNA stemness score in 18 types of cancer. **(B)** The DNA stemness score difference among CAPER score groups. **(C)** The results of the Spearman correlation between single gene expression and RNA stemness score in 18 types of cancer. **(D)** The RNA stemness score difference among CAPER score groups.
